# Effects of Bullying on Anxiety, Depression, and Posttraumatic Stress Disorder Among Sexual Minority Youths: Network Analysis

**DOI:** 10.2196/47233

**Published:** 2023-11-01

**Authors:** Jiaqi Li, Yu Jin, Shicun Xu, Amanda Wilson, Chang Chen, Xianyu Luo, Yuhang Liu, Xi Ling, Xi Sun, Yuanyuan Wang

**Affiliations:** 1 School of Psychology South China Normal University Guangzhou China; 2 Key Laboratory of Brain, Cognition and Education Sciences Ministry of Education Guangzhou China; 3 Center for Studies of Psychological Application South China Normal University Guangzhou China; 4 Guangdong Key Laboratory of Mental Health and Cognitive Science South China Normal University Guangzhou China; 5 College of Education for the Future Beijing Normal University Beijing China; 6 Northeast Asian Research Center Jilin University Changchun China; 7 Department of Population, Resources and Environment Jilin University Changchun China; 8 Faculty of Health and Life Sciences De Montfort University De Montfort United Kingdom

**Keywords:** sexual minority youths, bullying victimization, anxiety, depression, posttraumatic stress disorder, network analysis, Bayesian network

## Abstract

**Background:**

Bullying victimization is highly prevalent among sexual minority youths, particularly in educational settings, negatively affecting their mental health. However, previous studies have scarcely explored the symptomatic relationships among anxiety, depression, and posttraumatic stress disorder (PTSD) among sexual minority youths who experienced bullying on college campuses.

**Objective:**

The objectives of our study were to (1) characterize the anxiety-depression-PTSD network structures of gay or lesbian, bisexuals, and other sexual minority youths previously bullied on college campuses; and (2) compare symptomatic associations in the anxiety-depression-PTSD networks among bullied sexual minority youths and heterosexual youths’ groups.

**Methods:**

This cross-sectional study recruited college participants from Jilin Province, China. Data were analyzed using a subset of the data extracted after screening for sexual orientation and history of bullying victimization. Sexual minority youths were then divided into 3 subgroups: gay or lesbian (homosexual), bisexual, and other. Mental health symptom severity was assessed using scales: the 7-item Generalized Anxiety Disorder Scale measuring anxiety, the 9-item Patient Health Questionnaire measuring depression, and the 10-item Trauma Screening Questionnaire measuring PTSD symptoms. Combining the undirected and Bayesian network analyses, the anxiety-depression-PTSD networks were compared among sexual minority youths subgroups, and the difference between heterosexual youths and sexual minority youths was investigated. Chi-square tests were used to compare the difference in categorical variables, while independent-sample *t* tests were run on continuous variables.

**Results:**

In this large-scale sample of 89,342 participants, 12,249 identified as sexual minority youths, of which 1603 (13.1%, 95% CI 12.5%-13.7%) reported being bullied on college campuses in the past year. According to the expected influence (EI) and bridge expected influence (bEI) index, in the global network structure of anxiety, depression, and PTSD, sad mood (EI=1.078, bEI=0.635) and irritability (EI=1.077, bEI=0.954) were identified as central and bridge symptoms; emotional cue reactivity (EI=1.015) was a central symptom of PTSD in this global network. In the anxiety-depression-PTSD Bayesian network, anhedonia had the highest prediction priority for activating other symptoms; and feeling afraid linked symptoms from anxiety to the PTSD community. Compared to their heterosexual counterparts, sexual minority youths exhibited a stronger association between difficulty concentrating and appetite. The “sad mood-appetite” edge was strongest in the gay or lesbian network; the “irritability-exaggerated startle response” edge was strongest in the bisexual network.

**Conclusions:**

For the first time, this study identified the most central and bridge symptoms (sad mood and irritability) within the depression-anxiety-PTSD network of sexual minority youths with past bullying-victim experiences on college campuses. Emotional cue reactivity, anhedonia, and feeling afraid were other vital symptoms in the comorbid network. Symptomatic relationships existed showing heterogeneity in bullied heterosexual youths and sexual minority youth networks, which also was present within the sexual minority youth subgroups. Consequently, refined targeted interventions are required to relieve anxiety, depression, and PTSD symptoms.

## Introduction

Bullying is defined as the intent or behavior that seeks to harm another person repeatedly, with an imbalance of power [1,2]. It is reported in the literature that the rate of bullying in higher education (on college or university campuses) varies across cultures, ranging from 1.7% to 25.2% [3-5]. Particularly, sexual minority youths, who identify their sexual orientations as nonheterosexual, such as lesbian, gay, or bisexual, are vulnerable to discrimination and bullying [5,6], and have higher odds of experiencing bullying on campus than their heterosexual counterparts [7-10]. A national survey in the United States further reported that sexual minority youths experience a high rate of verbal harassment (76.1%), sexual harassment (53.7%), physical harassment (31.2%), and physical assault (12.5%) [11]. In addition, after being bullied, sexual minority youths may experience adverse physical health and persistent negative effects on their mental health [12].

A considerable amount of literature has reported that sexual minority youths exhibit higher rates of anxiety, depression, and posttraumatic stress disorder (PTSD) than their heterosexual counterparts [13-16]. As a recent meta-analysis noted [17], compared to heterosexual counterparts, lesbian or gay, as well as bisexual individuals yielded a higher risk of anxiety and depression. In addition, sexual minority individuals were found to be vulnerable to adverse experiences, such as being harassed and even bullied in school, increasing the risk for anxiety, depression, and PTSD than those participants who have not experienced adverse life events [18].

Past research has found differences in mental health problems among subgroups of sexual minorities [17,19]. For example, the result from a longitudinal study suggests that bullying victimization is connected to stronger depressive symptoms among bisexual women, and stronger anxiety symptoms among bisexual men [20]. Bisexual individuals are also more likely to report PTSD than their gay or lesbian counterparts [21]. Pansexual women appear to be more open about their sexuality but with a higher consciousness in regard to their sexual orientation-based stigma than bisexual women, which is linked to poorer mental health [22]. Further, asexual men are suggested to have higher scores of depression and psychiatric disturbance than their homosexual and bisexual counterparts [23]. The above studies show a high heterogeneity of mental health outcomes among sexual minority subgroups. However, whether these differences remain consistent among sexual minority youths who are being bullied on college campuses requires further exploration.

Minority stress theory provides a conceptual model that the mental health issues of sexual minorities are partially derived from minority stressors, which are defined as the excess stress to which individuals from stigmatized social categories are exposed to due to their minority position [24], such as higher rates of bullying and discrimination. Furthermore, minority stress can cause biological responses that are related to mental health symptoms [25]. For example, exposure to general minority stress can affect one’s gene expression, which is connected to cardiovascular function [26] and exerts an interactional effect between anxiety, depression, and PTSD [27-29]. Meanwhile, it may increase the dysregulation of the hypothalamus-pituitary-adrenal axis, directly adding to the risk of depression, anxiety, and PTSD [30,31]. Nevertheless, the symptomatic relationship between anxiety, depression, and PTSD among sexual minority youths who are bullied on college campuses lacks investigation.

In recent years, several published studies have adopted network analysis in both clinical psychology or psychiatric fields to explore the relationship between symptoms of various disorders [32-34]. The undirected network analysis depicts the interactions between individual symptoms, with a symptom being termed a “node,” and the partial correlation coefficient between 2 nodes determining their “edge” [35]. According to the expected influence (EI) index and the bridge expected influence (bEI) index, the central and bridge symptoms of the network structure could be identified, respectively [36,37]. Furthermore, in this study, Bayesian network analysis was applied to clarify the directed relationship between symptoms further. Based on the completed partially directed acyclic graph (CPDAG), Bayesian network analysis can unclose the putative directions of the potential causal relationship between 2 nodes [38], and it could help to discover activating paths among symptoms of anxiety, depression, and PTSD network [33].

Given these premises, this study was implemented using undirected and Bayesian network analyses to achieve 2 main objectives: first, to characterize the symptomatic relationships in the anxiety-depression-PTSD network among bullied sexual minority youths and sexual minority youth subgroups; second, to compare the network structures among bullied sexual minority youth subgroups. For aim 1, based on previous results [34,39,40], the researchers hypothesized that depressive and anxious symptoms would be the most central and bridge among all symptoms. For aim 2, considering sexual minority youth subgroups have various prevalence and symptomatic manifestations of anxiety, depression, and PTSD [14,21], the researchers hypothesized that different strengths of symptomatic associations would exist among sexual minority youth subgroups who had disclosed experiences of being bullied. In addition, the researchers aimed to enrich the field, as well as help offer targeted suggestions and interventions for specific symptoms associated with poor mental health.

## Methods

### Participants and Settings

This study was designed with a cross-sectional approach, recruiting College students from 63 Universities or Colleges in Jilin Province, China, from October to November 2021. Students completed an electronic questionnaire with a Quick Response code, delivered by their teachers with their classmates. The inclusion criteria for recruitment were (1) being 15 years old or older; (2) studying at universities or colleges in Jilin Province, China; (3) understanding the Chinese questionnaire and having the ability to provide informed consent.

### Ethical Considerations

Ethical approval for this study was granted by the Ethics Committee of Jilin University (NO20210929 [11 October 2021]), following the 1964 Helsinki Declaration and its amendments in 2013. Electronic informed consent was provided by all participants, with consent that their answers could be applied to any secondary analyses.

### Measurements

Participants’ sexual orientation was measured by a single-option question with 6 items: “Which of the following better describes your sexual orientation?” Except for heterosexuality, all other sexual orientations are combined to form the sexual minority youth group [41,42], which was then further divided into 3 subgroups, including homosexuality (gay or lesbian), bisexuality, and others (asexuality, pansexuality, and uncertain). Anxiety was measured by the 7-item Generalized Anxiety Disorder Questionnaire [43], depression by the 9-item Patient Health Questionnaire [44], and PTSD by the 10-item Trauma Screening Questionnaire [45]. The experience of being bullied during higher education was measured by a single question: “Have you ever been bullied on campus in the past year?” Details of measurements are presented in the Section S1 in Multimedia Appendix 1, Additional information for networks.

### Statistical Analysis

#### Descriptive Analysis

In total, 117,769 participants were recruited and 89,342 were included according to the criteria and completion of the questionnaire. A descriptive analysis was run on the subdata extracted from screening for sexual orientation and history of bullying victimization. In the data set of those who had been bullied on college campuses in the past, they were divided into groups of heterosexual and sexual minority youth groups. Sociodemographic variables included participants’ age, sex, residence, ethnicity, family type, current annual income, and whether they were an only child. The categorical variables in heterosexual or sexual minority youth groups were compared by chi-square tests, while continuous variables were compared by two-tailed independent-sample *t* tests.

#### Undirected Network Estimation

This study used R programming [46] to structure the undirected network, with the R package “qgraph” (R Foundation for Statistical Computing) to visualize the Graphical Gaussian Model [35]. The R package “mgm” assessed the predictability of a node, referring to the variance of a node that could be explained by all others [47]. The differences between the sexual minority youths and heterosexual network structures and among sexual minority youth subgroups were compared using the R package “Network Comparison Test” with 1000 permutations [48]. For more detail on the method, see Section S2 in Multimedia Appendix 1, Additional information for networks.

#### Bayesian Network Estimation

In this study, DAG was modelled by the R package “bnlearn,” with the hill-climbing algorithm [49]. To further obtain a clear direction among symptoms, the Markov equivalence classes of the DAG were drawn, which can be described uniquely by a CPDAG [50]. In CPDAG, all arrows of undirected edges can be invertible, while all directed edges cannot be converted into undirected edges. Finally, in the determined CPDAG, the direct activating paths in the Bayesian network are more conclusive. More details are shown in Section S2 in Multimedia Appendix 1, Additional information for networks.

## Results

### Descriptive Statistics

Table 1 presents the sociodemographic characteristics of participants. Within groups, 5063 (6%) heterosexual participants and 1603 (13.1%) sexual minority youth participants reported being bullied on college campuses in the past year. The prevalence of bullying among heterosexual youths was 6% (95% CI 5.9%-6.2%), and 13.1% (95% CI 12.5%-13.7%) among sexual minority youths. Furthermore, among gay participants, the prevalence of bullying was 19.9% (95% CI 17.1%-23.1%), 12.2% (95% CI 9.8%-15.1%) among lesbians, 15.6% (95% CI 13.6%-17.9%) among bisexual males, and 12% (95% CI 11.2%-12.9%) among bisexual females. The odds of being bullied among sexual minority males was 14.4% (95% CI 12.7%-16.2%), and 11.8% (95% CI 10.6%-13%) among sexual minority females.

**Table 1:** Sociodemographic characteristics of participants who had been bullied on college campuses.

		Heterosexuals (n=5063), n (%)	Sexual minority youths (n=1603), n (%)	Lesbian (n=77), n (%)	Gay (n=142), n (%)	Bisexuals (n=815), n (%)	Others (n=569), n (%)	Χ²^a^		Degrees of freedom (*df*)		*P* value	
**Sex**									94.2		1		<.001^b^
	Male	2444 (48.3)	552 (34.4)	N/A^c^	142 (100)	176 (21.6)	234 (41.1)						
	Female	2619 (51.7)	1051 (65.6)	77 (100)	N/A	639 (78.4)	335 (58.9)						
**Residence**									44.6		1		<.001^b^
	City	2513 (49.6)	949 (59.2)	51 (66.2)	71 (50)	494 (60.6)	333 (58.5)						
	Town and county	2550 (50.4)	654 (40.8)	26 (33.8)	71 (50)	321 (39.4)	236 (41.5)						
**Ethnicity**									1.0		1		.31
	Han	4543 (89.7)	1424 (88.8)	74 (96.1)	119 (83.8)	728 (89.3)	503 (88.4)						
	Others	520 (10.3)	179 (11.2)	3 (3.9)	23 (16.2)	87 (10.7)	66 (11.6)						
**Family type**									17.2		2		<.001^b^
	Nuclear family	3311 (65.4)	1005 (62.7)	42 (54.5)	86 (60.6)	502 (61.6)	375 (65.9)						
	Above 3 generation	951 (18.8)	274 (17.1)	12 (15.6)	26 (18.3)	143 (17.5)	93 (16.3)						
	Others	801 (15.8)	324 (20.2)	23 (29.9)	30 (21.1)	170 (20.9)	101 (17.8)						
**Current annual income (US $)**									20.0		3		<.001^b^
	<930	1659 (32.8)	516 (32.2)	27 (35)	47 (33.1)	234 (28.7)	208 (36.6)						
	930-2169	1688 (33.3)	463 (28.9)	16 (20.8)	33 (23.2)	247 (30.3)	167 (29.3)						
	2170-3565	763 (15.1)	251 (15.6)	14 (18.2)	27 (19)	143 (17.5)	67 (11.8)						
	≥3565	953 (18.8)	373 (23.3)	20 (26)	35 (24.6)	191 (23.4)	127 (22.3)						
**Only-child status**									19.4		1		<.001^b^
	Yes	2340 (46.2)	842 (52.5)	42 (54.5)	72 (50.7)	408 (50.1)	320 (56.2)						
	No	2723 (53.8)	761 (47.5)	35 (45.5)	70 (49.3)	407 (49.9)	249 (43.8)						
Age (years), mean (SD)		19.54 (1.72)	19.57 (1.67)	19.55 (1.66)	19.74 (1.70)	19.51 (1.63)	19.63 (1.73)	–0.7^d^		6659		.49	
GAD-7^e^, mean (SD)		6.25 (474)	7.82 (5.44)	8.06 (5.90)	7.61 (5.30)	7.93 (5.15)	7.69 (5.80)	–11.2^d^		6664		<.001^b^	
PHQ-9^f^, mean (SD)		8.06 (5.30)	10.15 (6.33)	10.74 (7.52)	9.45 (5.42)	10.16 (5.96)	10.23 (6.85)	–13.1^d^		6664		<.001^b^	
PTSD-10^g^, mean (SD)		4.88 (3.16)	5.75 (3.12)	5.94 (3.38)	5.58 (3.20)	5.91 (2.97)	5.55 (3.27)	–9.7^d^		6664		<.001^b^	

^a^Х²: used to compare differences between heterosexual and sexual minority youth groups among specified demographic variables.

^b^There was a significant difference existing with *P*<.001.

^c^N/A: Not applicable.

^d^T: used to compare differences between heterosexual and sexual minority youth groups among specified demographic variables.

^e^GAD-7: the 7-item Generalized Anxiety Disorders Scale.

^f^PHQ-9: the 9-item Patient Health Questionnaire.

^g^PTSD-10: measured by the 10-item Trauma Screening Questionnaire.

Compared to their heterosexual counterparts, more sexual minority females, and more sexual minority youths living in urban residences reported being bullied on the campuses. In addition, experiences of being bullied were more likely to occur among participants from nuclear families, with lower annual family income, or among those who had siblings. Bullied sexual minority youths also reported higher rates of anxiety, depression, and PTSD symptoms than bullied heterosexual youths.

### Undirected Network Structure

**Figure 1:**
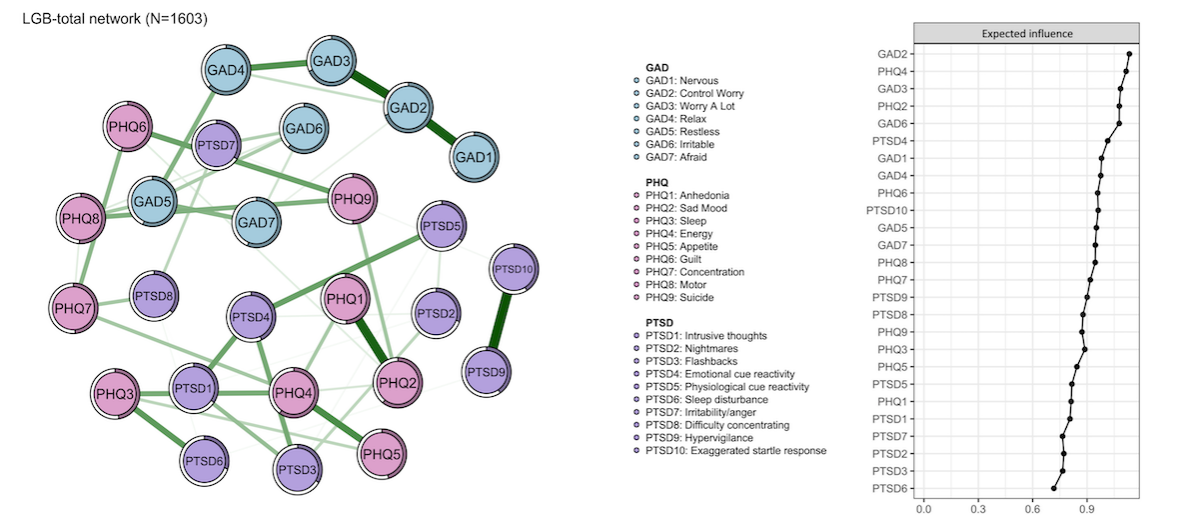
The global network structure of central symptoms among all bullied sexual minority youths (N=1603). GAD: the 7-item Generalized Anxiety Disorders Scale; LGB: homosexuality (gay or lesbian), bisexuality and other sexual minority youths; PHQ: the 9-item Patient Health Questionnaire; PTSD: posttraumatic stress disorder measured by the 10-item Trauma Screening Questionnaire.

As seen in Figure 1, according to the centrality index EI, GAD2 (Generalized Anxiety Disorders Scale; “control worry,” EI=1.134) had the highest EI, followed by PHQ4 (Patient Health Questionnaire; “energy,” EI=1.116), GAD3 (“worry a lot,” EI=1.085), PHQ2 (“sad mood,” EI=1.078), GAD6 (“irritability,” EI=1.077), and PTSD4 (“energy,” EI=1.015). These 6 symptoms played the most central role in the global network of anxiety, depression, and PTSD symptoms among all bullied sexual minority youths. The central network structures of the 3 sexual minority youth subgroups and their centrality index EI are shown in Table S1 and Figures S1, S2, S3, and S4 in Multimedia Appendix 1, Additional information for networks.

**Figure 2:**
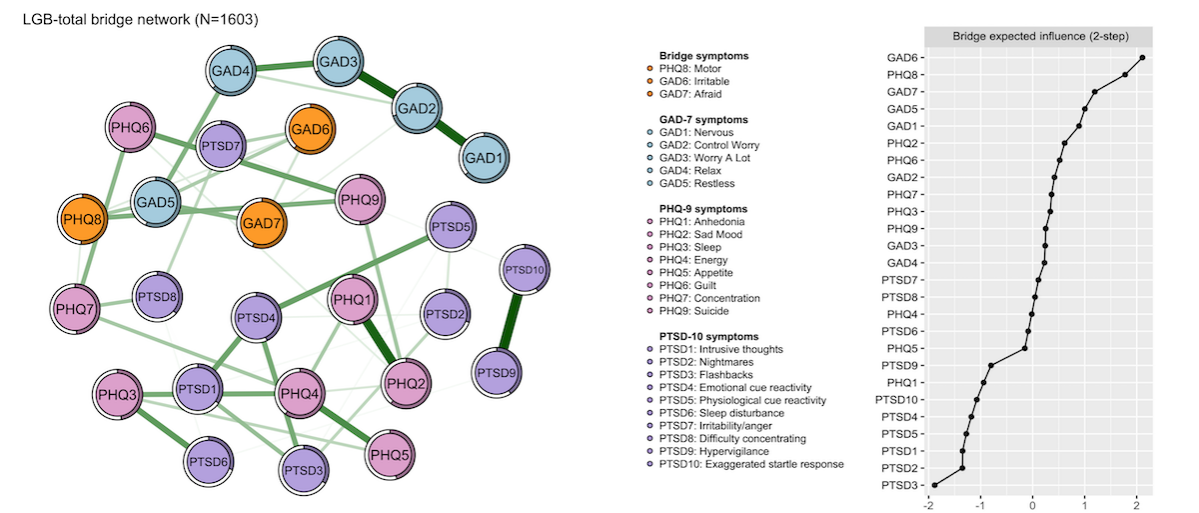
The global network structure of bridge symptoms among all bullied sexual minority youths (N=1603). GAD: the 7-item Generalized Anxiety Disorders Scale; LGB: homosexuality (gay or lesbian), bisexuality and other sexual minority youths; PHQ: the 9-item Patient Health Questionnaire; PTSD: posttraumatic stress disorder measured by the 10-item Trauma Screening Questionnaire.

As seen in Figure 2, according to bEI, GAD6 (“irritability,” bEI=0.954), PHQ8 (“motor,” bEI=0.883), GAD7 (“feeling afraid,” bEI=0.758), GAD5 (“restless,” bEI=0.718), GAD1 (“nervous,” bEI=0.694), and PHQ2 (“sad mood,” bEI=0.635) had crucial bridging roles in connecting to the other symptoms of anxiety, depression, or PTSD, which may possibly transform from 1 community to another. The bridge network structures of the 3 sexual minority youth subgroups and their bridge bEI index are shown in Table S1 and Figures S5, S6, S7, and S8 in Multimedia Appendix 1, Additional information for networks.

The average predictability of nodes was 48.67%, which means that, on average, the variance of 48.67% per node could be explained by its neighbors. In addition, all networks show good stability in this study (see in Figures S9, S10, S11, and S12 in Multimedia Appendix 1, Additional information for networks).

### Bayesian Network Structure

**Figure 3:**
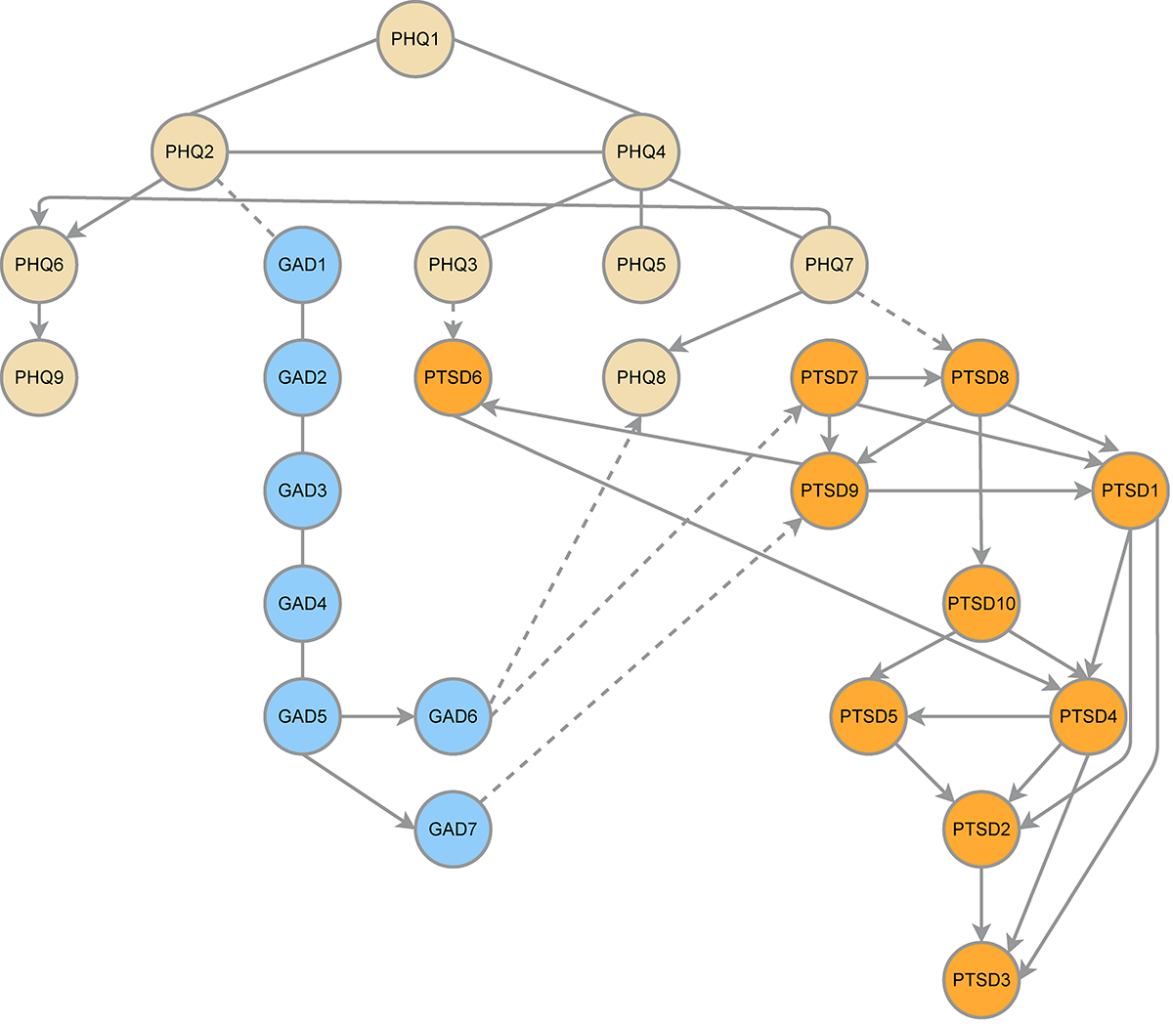
The Bayesian network of anxiety, depression, and posttraumatic stress disorder among bullied sexual minority youths, based on a completed partially directed acyclic graph. GAD: the 7-item Generalized Anxiety Disorders Scale; PHQ; the 9-item Patient Health Questionnaire; PTSD: posttraumatic stress disorder measured by the 10-item Trauma Screening Questionnaire. The edges without directions refer to reversiblity. The solid edges with direction refer to irreversiblity. The dotted edges with direction refer to irreversibility and arrowing to a node in another symptom community.

Figure 3 shows the CPDAG results, indicating that PHQ1 (“anhedonia”) had the highest predictive priority for other symptoms occurring in the anxiety-depression-PTSD network. PHQ2 (“sad mood”), GAD6 (“irritability”), and GAD7 (“feeling afraid”) were identified as 3 crucial bridge symptoms, whose downstream symptoms had more branches and could activate more pathways. In addition, there were reversible connections between PHQ1 (“anhedonia”), PHQ2 (“sad mood”), and PHQ4 (“appetite”). Whereas, the edges between PHQ2 (“sad mood”) and PHQ6 (“guilt”), between GAD6 (“irritability”), PHQ8 (“motor”), and PTSD7 (“irritability or anger”), and between GAD7 (“feeling afraid”) to PTSD9 (“hypervigilance”) were unidirectional and irreversible. These results indicate the most likely direction from both depression and anxiety symptoms to PTSD symptoms, rather than vice versa. More results of CPDAG can be found in Figure S13 in Multimedia Appendix 1, Additional information for networks.

### Network Comparison Structures

**Figure 4:**
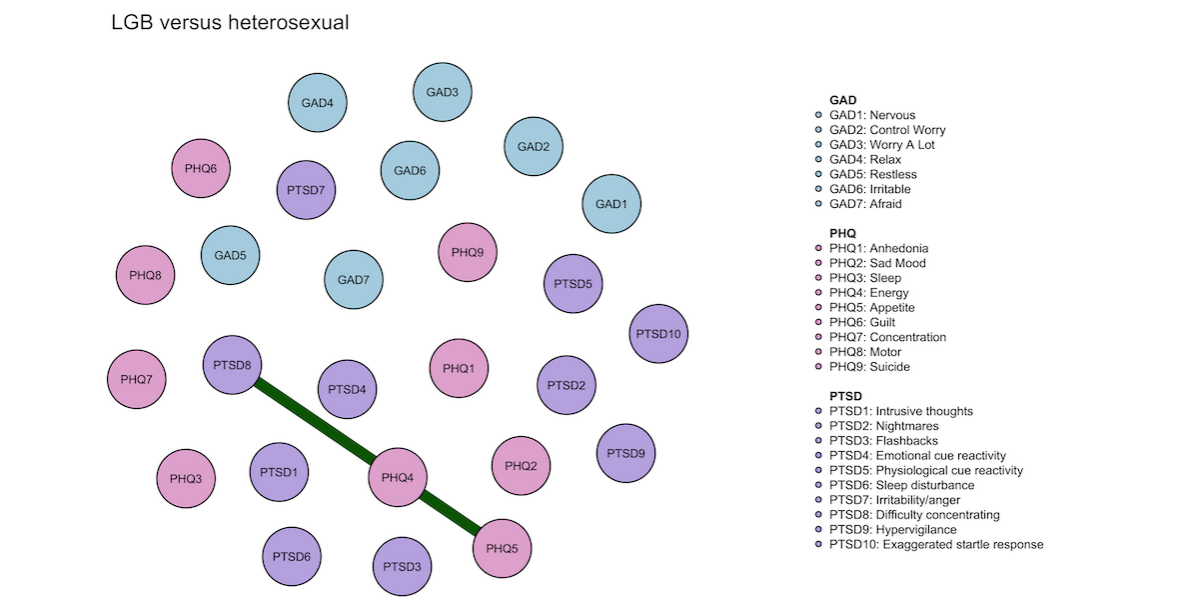
The comparison of network structure between sexual minority and heterosexual youths. GAD: the 7-item Generalized Anxiety Disorders Scale; PHQ: the 9-item Patient Health Questionnaire; PTSD: posttraumatic stress disorder measured by the 10-item Trauma Screening Questionnaire.

Figure 4 shows the results when comparing the network structures between the sexual minority youths and heterosexual groups. The edge of PTSD8 (“difficulty concentrating”)-PHQ5 (“appetite”) was significantly stronger in the sexual minority youth network structure than in the heterosexual.

Among the 3 subgroups of sexual minority youths, there was no significant difference in edges between the gay or lesbian network and the other sexual minority youth networks. However, the edge of PHQ2 (“sad mood”)-PHQ5 (“appetite”) was strongest in the gay or lesbian network and the edge of PTSD7 (“irritability”)-PTSD10 (“exaggerated startle response”) was strongest in the bisexual sexual minority youth networks (Figure S14 in Multimedia Appendix 1, Additional information for networks).

## Discussion

### Principal Findings

This is the first study based on a large-scale sample to investigate the symptomatic relationship of anxiety, depression, and PTSD among sexual minority youths bullied on college campuses in the past. The results partly supported hypothesis 1: sad mood and irritability were identified as the most frequently crucial activating symptoms of poor mental health, where emotional cue reactivity was the most central symptom of the PTSD community. In the Bayesian network, anhedonia had the highest predictive priority to activate the occurrence of all other symptoms; feeling afraid played a bridging role in the anxiety-depression-PTSD network. The results supported hypothesis 2: compared to heterosexuals, sexual minority youths exhibited a stronger association between difficulty concentrating and appetite. The “sad mood-appetite” edge was strongest in the gay or lesbian network, and the “irritability-exaggerated startle response” edge was strongest in the bisexual network.

Sexual minority youths has been frequently reported to experience discrimination, prejudice, and stigma-bias because of their sexual orientation [6,51], associated with bullying victimization of sexual minority youths [52,53]. Our study further reported a high prevalence of being bullied on college campuses among sexual minority youths, which was 13.3% (95% CI 12.5%-13.7%, n=1603). Consistent with previous findings, exposure to bullying on college campuses was found to be related to the onset of mental health problems among sexual minority youths [53]. As a meta-analysis supported, individuals who experience bullying victimization had increased odds of depression, anxiety, and PTSD [54].

Sad mood and irritability were the most crucial symptoms (both central and bridge) in all anxiety-depression-PTSD network models. Previous network analyses have consistently identified sad mood as the central and bridge symptom in the structure of the anxiety-depression network [36,55]. This result may be explained by the external stressors that sexual minority youths face, making them feel sad [51,56]. According to a US national survey on youths’ risk behavior, high rates of in-school bullying victimization for homosexuals (28.22%, n=91) and bisexuals (34.03%, n=318) occurred because of external discrimination [7]. When under stressful conditions, if those bullied students experience insufficient support from teachers, they could feel abandoned and hopeless [57]. In terms of irritability, 2 prior studies also noted that irritability had a high betweenness index (another bridge centrality index) [34], and was identified as one of the central symptoms in the depression-anxiety-PTSD network [58]. Research has indicated that, once bullied, an inability to find a coping strategy or resist bullying behaviors can make individuals feel frustrated, which may trap them in rumination anger [9]. Moreover, traumatic memories of bullying victimization could generate severe distress and irritability as well [59]. Our results showed irritability also had a high predictive priority of the occurrence of PTSD symptoms. Findings indicate that for those who do withstand cumulative bullying victimization, their subjective aggression can be triggered, potentially increasing their irritable mood and PTSD symptoms simultaneously [60]. Notably, emotional cue reactivity (in this study referring to feeling upset by reminders of the events) was the most central symptom in the PTSD network. This may be explained by the influences of autobiographical memories (a mixed memory of complicated life events) [61]. Experiences of being bullied incur negative emotional cues, such as insults and physical aggression [62], and these negative memories would, in turn, cause further distress.

In the Bayesian network, anhedonia had the highest predictive priority of activating other symptoms. It parallels a previous network analysis that identified anhedonia as a central symptom in the anxiety-depression network [36]. Previous findings reported that bullying behaviors could lead to poorer mental health for bullying victims [63]. After being bullied, sexual minority youths may form a negative schema about an institution, which influences their interaction with others, decreasing their interest in participating in activities with their peers [64]. Another possible explanation could be a lack of support from teachers and families after exposure to being bullied, which further hinders sexual minority youths’ mental health [65] and also makes it harder for them to perceive pleasure [66]. Additionally, being bullied may negatively influence the release of dopamine, a chemical produced by neuronal receptors that relate to feeling good, in the case of bullying, this causes a lack of pleasure [67]. Feeling afraid also played a significant bridging role in the Bayesian network, pointing to the occurrence of hypervigilance in the PTSD community. On the one hand, past bullying victimization could lead sexual minority youths to be afraid of reoccurring bullying [68]; on the other hand, without efficient support from others, exposing perpetrators of bullying could trigger their fear of retaliation [69]. In addition, feeling afraid is linked to the symptoms of PTSD in this study. As early findings stated, those bullied could incur idiopathic nightmares and sleep problems, leading to hypervigilance [70]. From the neuropsychological perspective, fear-related threats increase the steady-state visual evoked amplitudes, thus heightening individuals’ vigilance [71]. Based on this fear of repeated bullying, sexual minority youths’ visual attention to possible danger may present with over-heightened reactions.

Compared to the heterosexual network structure, difficulty concentrating was found to be more closely associated with appetite in the sexual minority youth network structure. As previous findings stated, sexual minority youths were more vulnerable to weight-based bullying by peers than heterosexual youths [72,73]. Moreover, a motivation to avoid or prevent bullying may be a key driver for those who seek treatment for obesity [74]. Thus, the ideal body image and weight bias may result in more sexual minority youths choosing to diet in order to lose weight or engage in harmful weight-control strategies, such as purging, to maintain a satisfactory appearance [75]. It should be noted that over-preoccupation with body shape might impair concentration in other areas, supported by a previous study in which those who experienced bullying reported that they perceived their attention was distracted due to the fear associated with the experience, which led to difficulty concentrating when learning and during academic achievement, further hindering their state of mental well-being [76].

Differently, compared to bullied bisexual youths, sad mood had a stronger correlation with appetite among bullied gay or lesbian youths. Researchers consistently report more disordered eating behaviors in gay or lesbian youths than in bisexuals [77]. In addition, gay or lesbian youths were reported to have more body dissatisfaction, driven by an ideal body shape [78,79]. Negative mood also relates to subjective appetite, causing poor appetite or overeating [80]. Since discrimination and bullying incur a sad mood for gay or lesbian youths, they may choose binge eating to elevate their mood momentarily [81]. Furthermore, compared to other sexual minority youths, bisexual youths exhibited a stronger correlation between irritability and exaggerated startle response. This may be due to bisexual youths experiencing dual discrimination from homosexual and heterosexual communities [82]. Since bullying shapes a threatening context for bisexual youths, they may become irritable and exhibit a high startle reactivity [83]. Meanwhile, higher irritability can alter attention bias, with decreased functional connectivity between the left inferior frontal gyrus and a periaqueductal grey area, which may increase the response to frustrating stimuli [84].

### Limitations

Several limitations should be noted in this study. First, as a cross-sectional study, it is hard to explore the dynamic process of psychopathological networks [85], thus, further longitudinal research is required to obtain causal conclusions. Second, the replicability and generalizability of network structures are still limited across various samples [86]. In the future, neuroimaging studies are required to explore the organic effects on the brain. Third, measurements were limited in several aspects: (1) the minority stressors, such as lacking peers or family support, were not directly measured, and the results could be biased because of other potential influences. (2) There may be a part of the youths who have hesitancy toward or concealment of being identified as sexual minority youths for fear of being exposed in the context. (3) In addition, the interaction of gender identity and sexual orientation should be considered in future investigations. For example, a transgender man attracted to a cisgender man could also consider himself gay. Further studies should be conducted covering diverse gender identities in detail. (4) Because the experience of being bullied on college campuses in the past was measured by a self-report as well, in which the standard to determine the conceptualization of bullying varies across individuals, it was inevitable to include samples with different severities of bullying victimization. Future research should provide more detailed standards to define bullying and explore the underlying mechanism between various types or levels of bullying behaviors and the impact this has on the mental health of sexual minority populations. Bullying off-campus perpetrated by peers should be measured, and its impacts must be explored. (5) The bias of recall errors or social desirability effects is unavoidable for data from subjective reporting scales. Finally, other psychiatric disorders, such as obsessive-compulsive disorder or eating disorders, were not measured directly, which may affect the results.

### Conclusions

This cross-sectional study based on a large-scale sample, combined the undirected network analysis and Bayesian network analysis, for the first time, identifying the most central and bridge symptoms (sad mood and irritability), as well as the central role of emotional cue reactivity within the depression-anxiety-PTSD network of sexual minority youths who were bullied on college campuses in the past. CPDAG results also indicated the vital roles of anhedonia and feeling afraid in the global Bayesian network in this study. In addition, the comparisons of networks supported three findings: (1) compared to heterosexuals, the edge of “difficulty concentrating” was stronger than in the global sexual minority youth network, (2) the edge of “sad mood-appetite” was strongest in the gay or lesbian network, and (3) the edge of “irritability-exaggerated startle response” was strongest in the bisexual network. These findings should be considered when offering targeted support for improving the physical health and mental well-being of sexual minority youths. Consequently, refined targeted interventions could be formulated [6] to relieve the symptoms of anxiety, depression, and PTSD comorbidity among sexual minority youths.

## Appendix

Multimedia Appendix 1 Additional information for networks.

## Abbreviations

bEI: bridge expected influence
CPDAG: completed partially directed acyclic graph
EI: expected influence
GAD: Generalized Anxiety Disorder Scale
PHQ: Patient Health Questionnaire
PTSD: posttraumatic stress disorder

## Data Availability

The data set for this speciﬁc paper is available from the corresponding author upon reasonable request.

## References

[1] Smith PK (2016). Bullying: definition, types, causes, consequences and intervention. Soc Personal Psychol Compass.

[2] Younan B (2019). A systematic review of bullying definitions: how definition and format affect study outcome. J Aggress Confl Peace Res.

[3] Lund EM, Ross SW (2017). Bullying perpetration, victimization, and demographic differences in college students: a review of the literature. Trauma Violence Abuse.

[4] Pörhölä M, Cvancara K, Kaal E, Kunttu K, Tampere K, Torres MB (2019). Bullying in university between peers and by personnel: cultural variation in prevalence, forms, and gender differences in four countries. Soc Psychol Educ.

[5] Sinkkonen HM, Puhakka H, Meriläinen M (2012). Bullying at a university: students' experiences of bullying. Studies in Higher Education.

[6] Smith TE, Bauerband LA, Aguayo D, McCall CS, Huang FL, Reinke WM, Herman KC (2022). School bullying and gender minority youth: victimization experiences and perceived prevalence. School Psych Rev.

[7] Angoff HD, Barnhart WR (2021). Bullying and cyberbullying among LGBQ and heterosexual youth from an intersectional perspective: findings from the 2017 national youth risk behavior survey. J Sch Violence.

[8] Wensley K, Campbell M (2012). Heterosexual and nonheterosexual young university students' involvement in traditional and cyber forms of bullying. Cyberpsychol Behav Soc Netw.

[9] Zsila Á, Urbán Róbert, Demetrovics Z (2018). Anger rumination and unjust world beliefs moderate the association between cyberbullying victimization and psychiatric symptoms. Psychiatry Res.

[10] Kann L, McManus T, Harris WA, Shanklin SL, Flint KH, Queen B, Lowry R, Chyen D, Whittle L, Thornton J, Lim C, Bradford D, Yamakawa Y, Leon M, Brener N, Ethier KA (2018). Youth risk behavior surveillance - United States, 2017. MMWR Surveill Summ.

[11] Kosciw JG, Clark CM, Menard L (2022). The 2021 National School Climate Survey: The experiences of LGBTQ+ youth in our nation's schools.

[12] Russell ST, Fish JN (2016). Mental health in lesbian, gay, bisexual, and transgender (LGBT) youth. Annu Rev Clin Psychol.

[13] Mittleman J (2019). Sexual minority bullying and mental health from early childhood through adolescence. J Adolesc Health.

[14] Ross LE, Salway T, Tarasoff LA, MacKay JM, Hawkins BW, Fehr CP (2018). Prevalence of depression and anxiety among bisexual people compared to gay, lesbian, and heterosexual individuals: a systematic review and meta-analysis. J Sex Res.

[15] Poteat VP, O'Brien MD, Rosenbach SB, Finch EK, Calzo JP (2021). Depression, anxiety, and interest in mental health resources in school-based gender-sexuality alliances: implications for sexual and gender minority youth health promotion. Prev Sci.

[16] Burton CM, Marshal MP, Chisolm DJ, Sucato GS, Friedman MS (2013). Sexual minority-related victimization as a mediator of mental health disparities in sexual minority youth: a longitudinal analysis. J Youth Adolesc.

[17] Wittgens C, Fischer MM, Buspavanich P, Theobald S, Schweizer K, Trautmann S (2022). Mental health in people with minority sexual orientations: A meta-analysis of population-based studies. Acta Psychiatr Scand.

[18] Schnarrs PW, Stone AL, Bond MA, Salcido R, Dorri AA, Nemeroff CB (2022). Development and psychometric properties of the sexual and gender minority adverse childhood experiences (SGM-ACEs): Effect on sexual and gender minority adult mental health. Child Abuse Negl.

[19] Borgogna NC, McDermott RC, Aita SL, Kridel MM (2019). Anxiety and depression across gender and sexual minorities: implications for transgender, gender nonconforming, pansexual, demisexual, asexual, queer, and questioning individuals. Psychol Sex Orientat Gend Divers.

[20] Katz-Wise SL, Rosario M, Calzo JP, Scherer EA, Sarda V, Austin SB (2017). Associations of timing of sexual orientation developmental milestones and other sexual minority stressors with internalizing mental health symptoms among sexual minority young adults. Arch Sex Behav.

[21] Carey FR, LeardMann CA, Lehavot K, Jacobson IG, Kolaja CA, Stander VA, Rull RP (2022). Health disparities among lesbian, gay, and bisexual service members and veterans. Am J Prev Med.

[22] Morandini J, Strudwick J, Menzies R, Dar-Nimrod I (2022). Differences between Australian bisexual and pansexual women: an assessment of minority stressors and psychological outcomes. Psychol Sex.

[23] Yule MA, Brotto LA, Gorzalka BB (2013). Mental health and interpersonal functioning in self-identified asexual men and women. Psychol Sex.

[24] Meyer IH (2003). Prejudice, social stress, and mental health in lesbian, gay, and bisexual populations: conceptual issues and research evidence. Psychol Bull.

[25] Flentje A, Heck NC, Brennan JM, Meyer IH (2020). The relationship between minority stress and biological outcomes: a systematic review. J Behav Med.

[26] Flentje A, Kober KM, Carrico AW, Neilands TB, Flowers E, Heck NC, Aouizerat BE (2018). Minority stress and leukocyte gene expression in sexual minority men living with treated HIV infection. Brain Behav Immun.

[27] Dedert EA, Calhoun PS, Watkins LL, Sherwood A, Beckham JC (2010). Posttraumatic stress disorder, cardiovascular, and metabolic disease: a review of the evidence. Ann Behav Med.

[28] Gustad LT, Bjerkeset O, Strand LB, Janszky I, Salvesen Ø, Dalen H (2016). Cardiac function associated with previous, current and repeated depression and anxiety symptoms in a healthy population: the HUNT study. Open Heart.

[29] Johnson AK, Hayes SN, Sawchuk C, Johnson MP, Best PJ, Gulati R, Tweet MS (2020). Analysis of posttraumatic stress disorder, depression, anxiety, and resiliency within the unique population of spontaneous coronary artery dissection survivors. J Am Heart Assoc.

[30] Almeida FB, Pinna G, Barros HMT (2021). The role of HPA axis and allopregnanolone on the neurobiology of major depressive disorders and PTSD. Int J Mol Sci.

[31] Hinds JA, Sanchez ER (2022). The role of the Hypothalamus–Pituitary–Adrenal (HPA) axis in test-induced anxiety: assessments, physiological responses, and molecular details. Stresses.

[32] Amore M, Murri MB, Calcagno P, Rocca P, Rossi A, Aguglia E, Bellomo A, Blasi G, Carpiniello B, Cuomo A, dell'Osso L, di Giannantonio M, Giordano GM, Marchesi C, Monteleone P, Montemagni C, Oldani L, Pompili M, Roncone R, Rossi R, Siracusano A, Vita A, Zeppegno P, Corso A, Arzani C, Galderisi S, Maj M (2020). The association between insight and depressive symptoms in schizophrenia: Undirected and Bayesian network analyses. Eur Psychiatry.

[33] McNally RJ, Heeren A, Robinaugh DJ (2017). A Bayesian network analysis of posttraumatic stress disorder symptoms in adults reporting childhood sexual abuse. Eur J Psychotraumatol.

[34] Price M, Legrand AC, Brier ZMF, Hébert-Dufresne Laurent (2019). The symptoms at the center: examining the comorbidity of posttraumatic stress disorder, generalized anxiety disorder, and depression with network analysis. J Psychiatr Res.

[35] Epskamp S, Borsboom D, Fried EI (2018). Estimating psychological networks and their accuracy: a tutorial paper. Behav Res Methods.

[36] Beard C, Millner AJ, Forgeard MJC, Fried EI, Hsu KJ, Treadway MT, Leonard CV, Kertz SJ, Björgvinsson T (2016). Network analysis of depression and anxiety symptom relationships in a psychiatric sample. Psychol Med.

[37] Jones PJ, Ma R, McNally RJ (2021). Bridge centrality: a network approach to understanding comorbidity. Multivariate Behav Res.

[38] Pearl J, Russell S (2011). Bayesian networks.

[39] Contreras A, Nieto I, Valiente C, Espinosa R, Vazquez C (2019). The study of psychopathology from the network analysis perspective: a systematic review. Psychother Psychosom.

[40] Jin Y, Xu S, Hu Z, Li J, Li H, Wang X, Sun X, Wang Y (2023). Co-occurrence of PTSD and affective symptoms in a large sample with childhood trauma subtypes: a network analysis. Front Public Health.

[41] Rice K (2015). Pansexuality. Int Encyclopedia Human Sexuality.

[42] Hsieh N, Shuster SM (2021). Health and health care of sexual and gender minorities. J Health Soc Behav.

[43] Spitzer RL, Kroenke K, Williams JBW, Löwe Bernd (2006). A brief measure for assessing generalized anxiety disorder: the GAD-7. Arch Intern Med.

[44] Kroenke K, Spitzer RL, Williams JB (2001). The PHQ-9: validity of a brief depression severity measure. J Gen Intern Med.

[45] Brewin CR, Rose S, Andrews B, Green J, Tata P, McEvedy C, Turner S, Foa EB (2018). Brief screening instrument for post-traumatic stress disorder. Br J Psychiatry.

[46] Ihaka R, Gentleman R (1996). R: a language for data analysis and graphics. J Comput Graph Stat.

[47] Haslbeck Jmb, Waldorp LJ (2020). mgm: estimating time-varying mixed graphical models in high-dimensional data. J Stat Soft.

[48] van Borkulo CD, van Bork R, Boschloo L, Kossakowski JJ, Tio P, Schoevers RA, Borsboom D, Waldorp LJ (2022). Comparing network structures on three aspects: a permutation test. Psychol Methods.

[49] Scutari M (2010). Learning bayesian networks with the bnlearn R package. J Stat Softw.

[50] Castelletti F, Consonni G, Della Vedova ML, Peluso S (2018). Learning markov equivalence classes of directed acyclic graphs: an objective bayes approach. Bayesian Anal.

[51] Ayhan CHB, Bilgin H, Uluman OT, Sukut O, Yilmaz S, Buzlu S (2020). A systematic review of the discrimination against sexual and gender minority in health care settings. Int J Health Serv.

[52] D'Urso G, Pace U (2019). Homophobic bullying among adolescents: the role of insecure-dismissing attachment and peer support. J LGBT Youth.

[53] Zhao M, Xiao D, Wang W, Wu R, Zhang W, Guo L, Lu C (2021). Association among maltreatment, bullying and mental health, risk behavior and sexual attraction in chinese students. Acad Pediatr.

[54] Moore SE, Norman RE, Suetani S, Thomas HJ, Sly PD, Scott JG (2017). Consequences of bullying victimization in childhood and adolescence: a systematic review and meta-analysis. World J Psychiatry.

[55] Kaiser T, Herzog P, Voderholzer U, Brakemeier EL (2021). Unraveling the comorbidity of depression and anxiety in a large inpatient sample: network analysis to examine bridge symptoms. Depress Anxiety.

[56] Bettis AH, Liu RT (2019). Population-based analysis of temporal trends in the prevalence of depressed mood among sexual minority and heterosexual youths from 1999 through 2017. JAMA Pediatr.

[57] Tholander M, Lindberg A, Svensson D (2019). “A freak that no one can love”: difficult knowledge in testimonials on school bullying. Res Pap.

[58] Ma S, Yang J, Xu J, Zhang N, Kang L, Wang P, Wang W, Yang B, Li R, Xiang D, Bai H, Liu Z (2022). Using network analysis to identify central symptoms of college students' mental health. J Affect Disord.

[59] Källmén Håkan, Hallgren M (2021). Bullying at school and mental health problems among adolescents: a repeated cross-sectional study. Child Adolesc Psychiatry Ment Health.

[60] Kircanski K, White LK, Tseng WL, Wiggins JL, Frank HR, Sequeira S, Zhang S, Abend R, Towbin KE, Stringaris A, Pine DS, Leibenluft E, Brotman MA (2018). A latent variable approach to differentiating neural mechanisms of irritability and anxiety in youth. JAMA Psychiatry.

[61] Sheldon S, Williams K, Harrington S, Otto AR (2020). Emotional cue effects on accessing and elaborating upon autobiographical memories. Cognition.

[62] Solomon DT, Combs EM, Allen K, Roles S, DiCarlo S, Reed O, Klaver SJ (2019). The impact of minority stress and gender identity on PTSD outcomes in sexual minority survivors of interpersonal trauma. Psychol Sex.

[63] Evans CBR, Smokowski PR, Rose RA, Mercado MC, Marshall KJ (2018). Cumulative bullying experiences, adolescent behavioral and mental health, and academic achievement: an integrative model of perpetration, victimization, and bystander behavior. J Child Fam Stud.

[64] Braun A, Santesteban-Echarri O, Cadenhead KS, Cornblatt BA, Granholm E, Addington J (2022). Bullying and social functioning, schemas, and beliefs among youth at clinical high risk for psychosis. Early Interv Psychiatry.

[65] Day JK, Fish JN, Grossman AH, Russell ST (2020). Gay-straight alliances, inclusive policy, and school climate: LGBTQ youths' experiences of social support and bullying. J Res Adolesc.

[66] Luk JW, Gilman SE, Haynie DL, Simons-Morton BG (2018). Sexual orientation and depressive symptoms in adolescents. Pediatrics.

[67] Ke T, De Simoni S, Barker E, Smith P (2022). The association between peer-victimisation and structural and functional brain outcomes: a systematic review. JCPP Adv.

[68] Barnett AP, Molock SD, Nieves-Lugo K, Zea MC (2019). Anti-LGBT victimization, fear of violence at school, and suicide risk among adolescents. Psychol Sex Orientat Gend Divers.

[69] Bjereld Y, Daneback K, Mishna F (2019). Adults’ responses to bullying: the victimized youth’s perspectives. Res Pap Educ.

[70] Nielsen T (2017). The stress acceleration hypothesis of nightmares. Front Neurol.

[71] Kastner-Dorn AK, Andreatta M, Pauli P, Wieser MJ (2018). Hypervigilance during anxiety and selective attention during fear: using steady-state visual evoked potentials (ssVEPs) to disentangle attention mechanisms during predictable and unpredictable threat. Cortex.

[72] Puhl RM, Lessard LM (2020). Weight stigma in youth: prevalence, consequences, and considerations for clinical practice. Curr Obes Rep.

[73] Bucchianeri MM, Gower AL, McMorris BJ, Eisenberg ME (2016). Youth experiences with multiple types of prejudice-based harassment. J Adolesc.

[74] Reece LJ, Bissell P, Copeland RJ (2016). 'I just don't want to get bullied anymore, then I can lead a normal life'; insights into life as an obese adolescent and their views on obesity treatment. Health Expect.

[75] Himmelstein MS, Puhl RM, Watson RJ (2019). Weight-based victimization, eating behaviors, and weight-related health in sexual and gender minority adolescents. Appetite.

[76] Al-Raqqad HK, Al-Bourini ES, Al Talahin FM, Aranki RME (2017). The impact of school bullying on students' academic achievement from teachers point of view. Int Educ Stud.

[77] Cao Z, Cini E, Pellegrini D, Fragkos KC (2023). The association between sexual orientation and eating disorders-related eating behaviours in adolescents: a systematic review and meta-analysis. Eur Eat Disord Rev.

[78] Yean C, Benau EM, Dakanalis A, Hormes JM, Perone J, Timko CA (2013). The relationship of sex and sexual orientation to self-esteem, body shape satisfaction, and eating disorder symptomatology. Front Psychol.

[79] Cella S, Iannaccone M, Ascione R, Cotrufo P (2010). Body dissatisfaction, abnormal eating behaviours and eating disorder attitude in homo- and heterosexuals. Eat Weight Disord.

[80] Hepworth R, Mogg K, Brignell C, Bradley BP (2010). Negative mood increases selective attention to food cues and subjective appetite. Appetite.

[81] Svaldi J, Werle D, Naumann E, Eichler E, Berking M (2019). Prospective associations of negative mood and emotion regulation in the occurrence of binge eating in binge eating disorder. J Psychiatr Res.

[82] Doan Van EE, Mereish EH, Woulfe JM, Katz-Wise SL (2019). Perceived discrimination, coping mechanisms, and effects on health in bisexual and other non-monosexual adults. Arch Sex Behav.

[83] Radoman M, Akinbo FD, Rospenda KM, Gorka SM (2019). The impact of startle reactivity to unpredictable threat on the relation between bullying victimization and internalizing psychopathology. J Psychiatr Res.

[84] Tseng WL, Deveney CM, Stoddard J, Kircanski K, Frackman AE, Yi JY, Hsu D, Moroney E, Machlin L, Donahue L, Roule A, Perhamus G, Reynolds RC, Roberson-Nay R, Hettema JM, Towbin KE, Stringaris A, Pine DS, Brotman MA, Leibenluft E (2019). Brain mechanisms of attention orienting following frustration: associations with irritability and age in youths. Am J Psychiatry.

[85] Ebner-Priemer UW, Eid M, Kleindienst N, Stabenow S, Trull TJ (2009). Analytic strategies for understanding affective (in)stability and other dynamic processes in psychopathology. J Abnorm Psychol.

[86] Forbes MK, Wright AGC, Markon KE, Krueger RF (2017). Evidence that psychopathology symptom networks have limited replicability. J Abnorm Psychol.

