# Supplementary material for: Effects of Bullying on Anxiety, Depression, and Posttraumatic Stress Disorder Among Sexual Minority Youths: Network Analysis
Source: JMIR Public Health Surveill. 2023 Nov 1;9:e47233. doi: 10.2196/47233 (PMC10652196; doi:10.2196/47233)
Supplement: Multimedia Appendix 1 [file publichealth_v9i1e47233_app1.docx]

**Multimedia Appendix 1**

Section S1. Measurements

Section S2. Undirected Network Estimation

Section S3. Bayesian Network Estimation

Table S1. The expected influence and bridge expected influence index in the global anxiety-depression-PTSD network structure among SMY youth.

Figure S1. The network structure of central symptoms of bullied homosexual (Gay/Lesbian) youth (N = 219).

Figure S2. The network structure of central symptoms of bullied bisexual youth (N = 815).

Figure S3. The network structure of central symptoms of bullied other sexual minority youth (N = 569).

Figure S4. Strength, betweenness, and closeness of networks of anxiety, depression, and post-traumatic stress disorder among all SMY and SMY subgroups.

Figure S5. The network structure of bridge symptoms of bullied homosexual (Gay/Lesbian) youth (N = 219).

Figure S6. The network structure of bridge symptoms of bullied bisexual youth (N = 815).

Figure S7. The network structure of bridge symptoms of bullied other sexual minority youth (N = 569).

Figure S8. Bridge strength, bridge betweenness, bridge closeness of networks of anxiety, depression, and post-traumatic stress disorder among all SMY and SMY subgroups.

Figure S9. The stability of centrality and bridge centrality indices of the global network using case-dropping bootstrap.

Figure S10. Bootstrapped confidence intervals of edge weights of the global network.

Figure S11. Estimation of edge weight difference of the global network by bootstrapped difference test.

Figure S12. Estimation of strength difference of the global network by bootstrapped difference test.

Figure S13. The Bayesian network of anxiety, depression, and post-traumatic stress disorder among bullied sexual minority youth based on a directed acyclic graph.

Figure S14. The comparison of network structures among homosexual (Gay/Lesbian), bisexual, and other sexual minority (Others) youth who were bullied on college campuses.

**Section S1. Measurements**

**Sexual Orientation**

Participants’ sexual orientation was measured by a single-option question: Which of the following better describes your sexual orientation? The options included: (1) Asexuality (lack of sexual attraction); (2) gay/lesbian (experience sexual attraction to the same gender); (3) heterosexuality (experience sexual attraction to the opposite gender); (4) bisexuality (experience sexual attraction to more than one gender); (5) pansexuality (attracted to any gender); (6) uncertain. Except for (3) heterosexuality, all other sexual orientations were termed SMY [1, 2]. The SMY group was then further divided into three subgroups, including homosexuality (gay/lesbian), bisexuality, and others (asexuality, pansexuality, and uncertain).

**Depression Symptoms**

The severity of depression was measured by The Chinese version of the nine-item Patient Health Questionnaire (PHQ-9) [3], demonstrating good reliability in the Chinese population [4]. It consists of nine items with a total score of 27. It has good internal reliability and criterion validity, both 0.86, with good sensitivity and specificity of 0.90 and 0.94, respectively [5].

**Anxiety Symptoms**

The severity of anxiety was measured by The Chinese version of the seven-item Generalized Anxiety Disorder Scale (GAD-7) [6]. It consists of seven items with a total score of 21. The Chinese version of the GAD-7 scale has an internal reliability of 0.90 and criterion validity of 0.86, with good sensitivity and specificity of 86.2% and 95.5%, respectively [7].

**PTSD Symptoms**

The severity of PTSD symptoms was measured by The ten-item Trauma Screening Questionnaire (TSQ-10) [8], adapted from the PTSD Symptom Scale-Self-Report Version (PSS-SR) [9]. The TSQ was examined with good sensitivity of 0.85 and a specificity of 0.89 [10], and acts as a validated screening tool for PTSD across different countries [11].

**Bullying victimization on college campuses**

The experience of being bullied on College or University campuses in the past was measured by a question with four options: “Have you ever been bullied on campus in the past year?” (1) Verbal bullying; (2) Physical violence; (3) Sexual harassment; (4) Not being bullied. These three major forms of bullying are commonly measured in previous studies on the mental health of youth in China [12].

**Section S2. Undirected Network Estimation**

Using R programming [13] to structure the undirected network, partial correlation coefficients of each two items (nodes), the edges of the network, and the Graphical Gaussian Model (GGM) (a network structure) were estimated. With the R package “qgraph”, the graphic least absolute shrinkage and selection operator (LASSO) and Extended Bayesian Information Criterion (EBIC) model were used to visualize the GGM [14]. The centrality of each node was ordered according to the expected influence index (EI), which was considered one of the superior centrality indices due to its consideration of negative edges. It measures the relative importance of a node, as the EI of a node represents the sum of all edge weights connecting to this node, with a higher EI value indicating a more central node [14, 15]. The bridge centrality of each node was ordered based on the bridge expected influence index (bEI) [16]. The R package “mgm” assessed the predictability of a node, referring to the variance of a node that could be explained by all others [17]. The network accuracy and stability were estimated by re-estimating cases, and evaluating the stability of EI centrality and the weight of the edges. With reducing and re-estimating cases, we investigated whether the order of the centrality index would change. The network accuracy and stability were estimated by re-estimating cases, and evaluating the stability of EI centrality and the weight of the edges. The correlation stability coefficient (CS-C), preferably above 0.5, was used to evaluate the stability of EI centrality [14]. The 95% non-parametric bootstrap confidence intervals (CIs) were used to estimate the weight of edges. Lower and narrow CIs indicated higher accuracy and stability. Finally, the difference in network properties was evaluated by the bootstrapped difference test.

The difference between the SMY and heterosexual network structures and among SMY subgroups were compared using the R package “Network Comparison Test” with 1,000 permutations [18]. First, the global network strength was compared according to the absolute values of all edge weights. Second, distributions of edge weights were compared to identify networks’ characteristics. Finally, each edge of all SMY subgroups was compared with correcting P values based on multiple Holm-Bonferroni trials.

**Section S3. Bayesian Network Estimation**

Directed acyclic graph (DAG) is an additional step in network analyses, based on the Bayesian method, combining DAG and a probability of distribution, the Bayesian network can be structured [19]. It was recently utilized to represent the causal relationship directions between pairs of nodes [20]. In this study, DAG was modelled by the R package “bnlearn” setting, with the hill-climbing algorithm [21]. It repeatedly computes new network models with iterations, while testing each model’s goodness of fit to the observed data according to the Bayesian information Criteria (BIC). It analyses one optimal model at a time from the proximity space of the current position, until it reaches a locally optimal model.

To further obtain a clear direction among symptoms, the Markov equivalence classes of the DAG were drawn, which can be described uniquely by a completed partially directed acyclic graph (CPDAG) [22]. The CPDAG includes directed and undirected edges. In CPDAG, all arrows of undirected edges can be invertible, while all directed edges cannot be converted into undirected edges. In addition, to determine whether an edge in a DAG is invertible, it is necessary to estimate whether it adds or changes a v-structure (e.g., GAD7→PTSD9←PTSD7) in the graph when converting the arrow direction between two nodes. If there is any change in the original v-structure after modification, that edge between two nodes is regarded as completely directed and irreversible [23]. Finally, in the determined CPDAG, the directing activating paths in the Bayesian network are more conclusive.

| Table S1. The expected influence and bridge expected influence index in the global anxiety-depression-PTSD network structure among SMY youth. | | | |
| --- | --- | --- | --- |
| Node | Symptoms | EI Value | bEI Value |
| GAD1 | Nervous | 0.980 | 0.694 |
| GAD2 | Control Worry | 1.134 | 0.592 |
| GAD3 | Worry A Lot | 1.085 | 0.554 |
| GAD4 | Relax | 0.976 | 0.552 |
| GAD5 | Restless | 0.952 | 0.718 |
| GAD6 | Irritable | 1.077 | 0.954 |
| GAD7 | Afraid | 0.946 | 0.758 |
| PHQ1 | Anhedonia | 0.812 | 0.301 |
| PHQ2 | Sad Mood | 1.078 | 0.635 |
| PHQ3 | Sleep | 0.888 | 0.575 |
| PHQ4 | Energy | 1.116 | 0.499 |
| PHQ5 | Appetite | 0.844 | 0.471 |
| PHQ6 | Guilt | 0.959 | 0.614 |
| PHQ7 | Concentration | 0.918 | 0.581 |
| PHQ8 | Motor | 0.945 | 0.883 |
| PHQ9 | Suicide | 0.872 | 0.557 |
| PTSD1 | Intrusive thoughts | 0.806 | 0.215 |
| PTSD2 | Nightmares | 0.772 | 0.214 |
| PTSD3 | Flash back | 0.766 | 0.100 |
| PTSD4 | Emotional cue reactivity | 1.014 | 0.251 |
| PTSD5 | Psychological cue reactivity | 0.816 | 0.230 |
| PTSD6 | Sleep disturbance | 0.717 | 0.485 |
| PTSD7 | Irritability/irritable | 0.765 | 0.527 |
| PTSD8 | Difficult concentrating | 0.878 | 0.513 |
| PTSD9 | Hypervigilance | 0.901 | 0.332 |
| PTSD10 | Exaggerated startle response | 0.962 | 0.273 |

Note: EI, expected influence; bEI, bridge expected influence; SMY, sexual minority youth; GAD, the seven-item Generalized Anxiety Disorders Scale; PHQ, the nine-item Patient Health Questionnaire; PTSD, measured by the ten-item Trauma Screening Questionnaire.

Figure S1. The network structure of central symptoms of bullied homosexual (Gay/Lesbian) youth (N = 219).


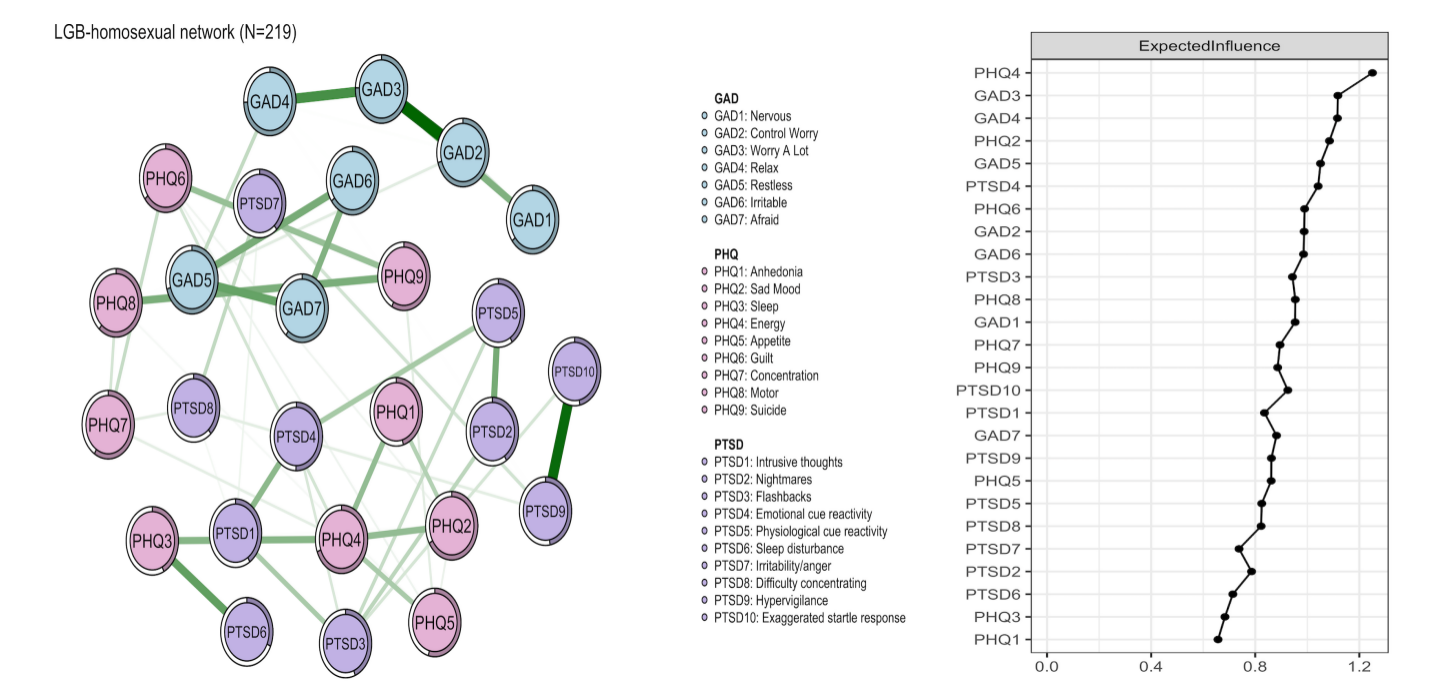


Note: GAD, the seven-item Generalized Anxiety Disorders Scale; PHQ, the nine-item Patient Health Questionnaire; PTSD, measured by the ten-item Trauma Screening Questionnaire.

Figure S2. The network structure of central symptoms of bullied bisexual youth (N = 815).


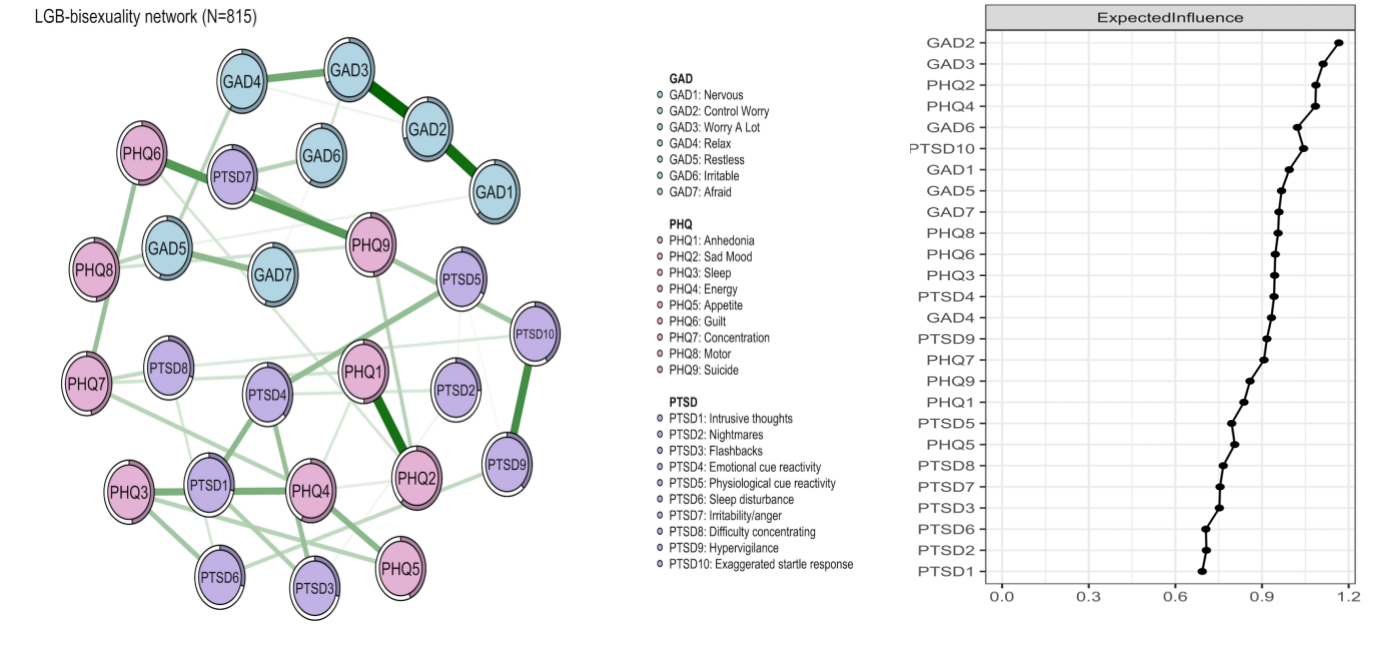


Note: GAD, the seven-item Generalized Anxiety Disorders Scale; PHQ, the nine-item Patient Health Questionnaire; PTSD, measured by the ten-item Trauma Screening Questionnaire.

Figure S3. The network structure of central symptoms of bullied other sexual minority youth (N = 569).


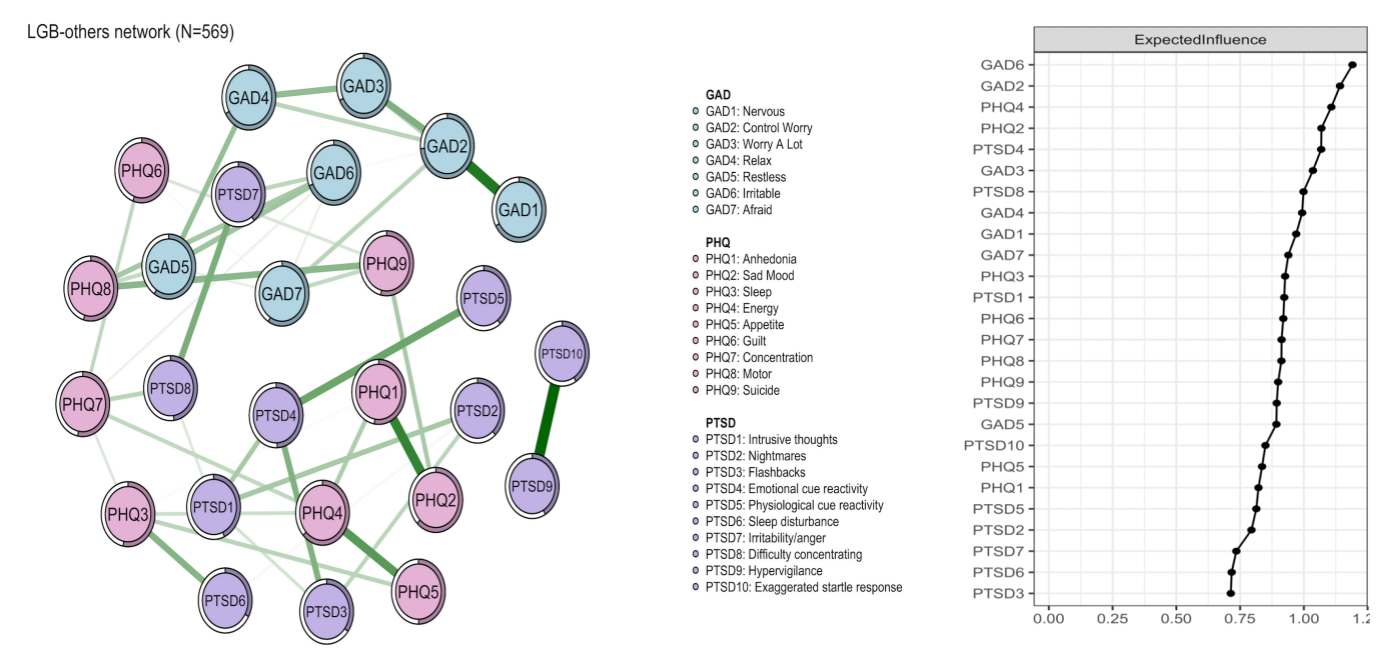


Note: GAD, the seven-item Generalized Anxiety Disorders Scale; PHQ, the nine-item Patient Health Questionnaire; PTSD, measured by the ten-item Trauma Screening Questionnaire.

Figure S4. Strength, betweenness, and closeness of networks of anxiety, depression, and post-traumatic stress disorder among all SMY and SMY subgroups.


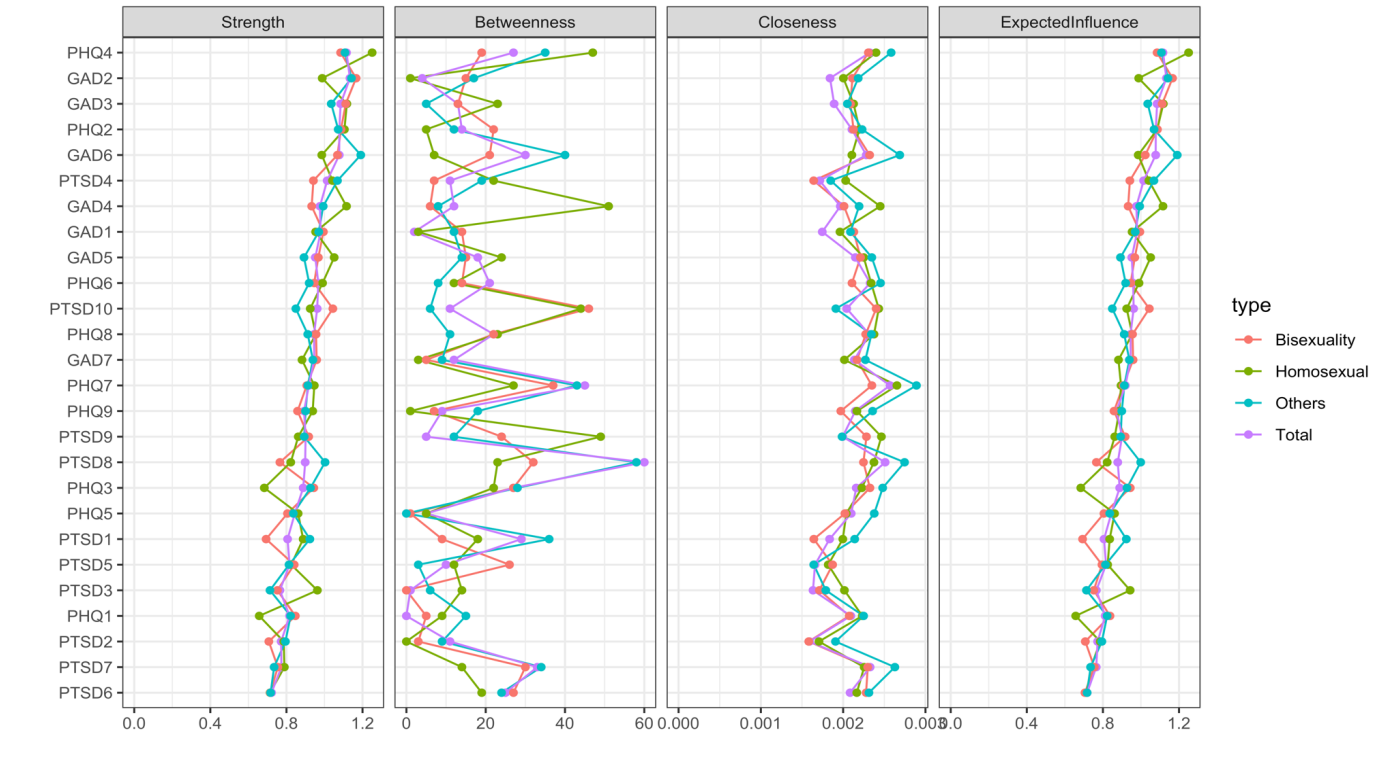


Note: SMY, sexual minority youth; GAD, the seven-item Generalized Anxiety Disorders Scale; PHQ, the nine-item Patient Health Questionnaire; PTSD, measured by the ten-item Trauma Screening Questionnaire.

Figure S5. The network structure of bridge symptoms of bullied homosexual (Gay/Lesbian) youth (N = 219).


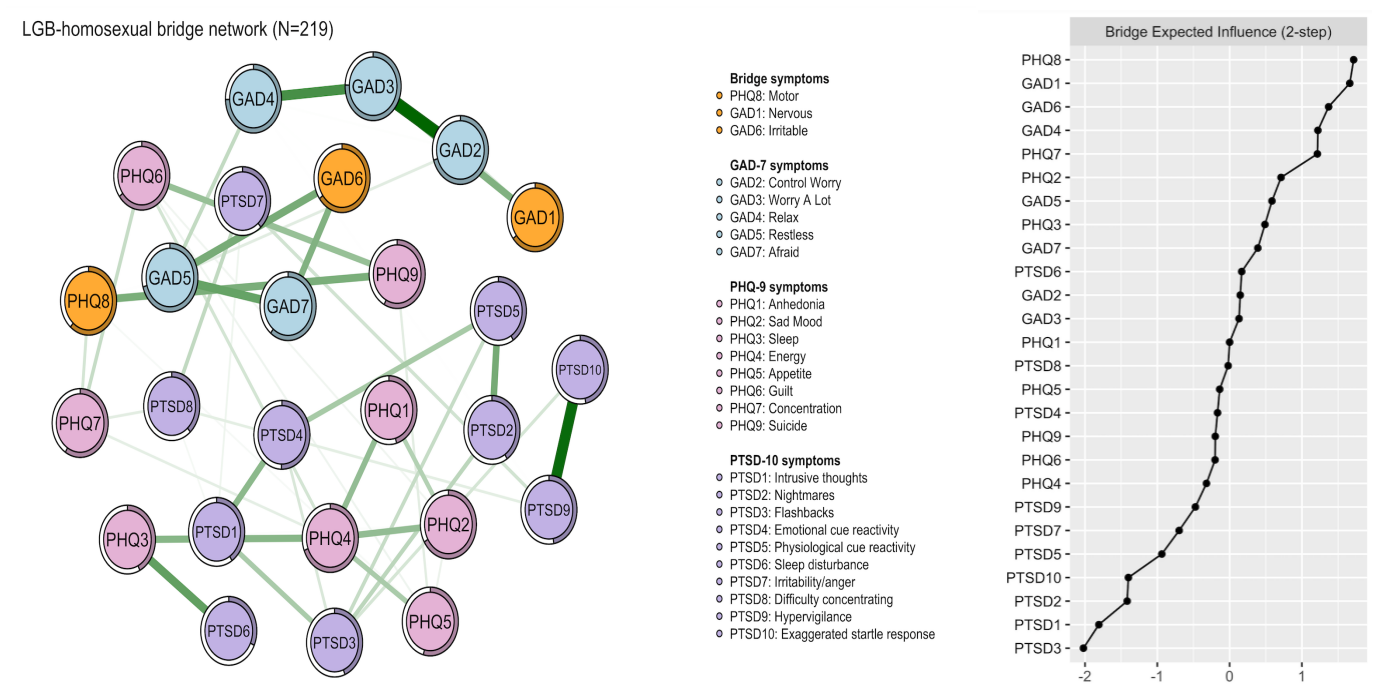


Note: GAD, the seven-item Generalized Anxiety Disorders Scale; PHQ, the nine-item Patient Health Questionnaire; PTSD, measured by the ten-item Trauma Screening Questionnaire.

Figure S6. The network structure of bridge symptoms of bullied bisexual youth (N = 815).


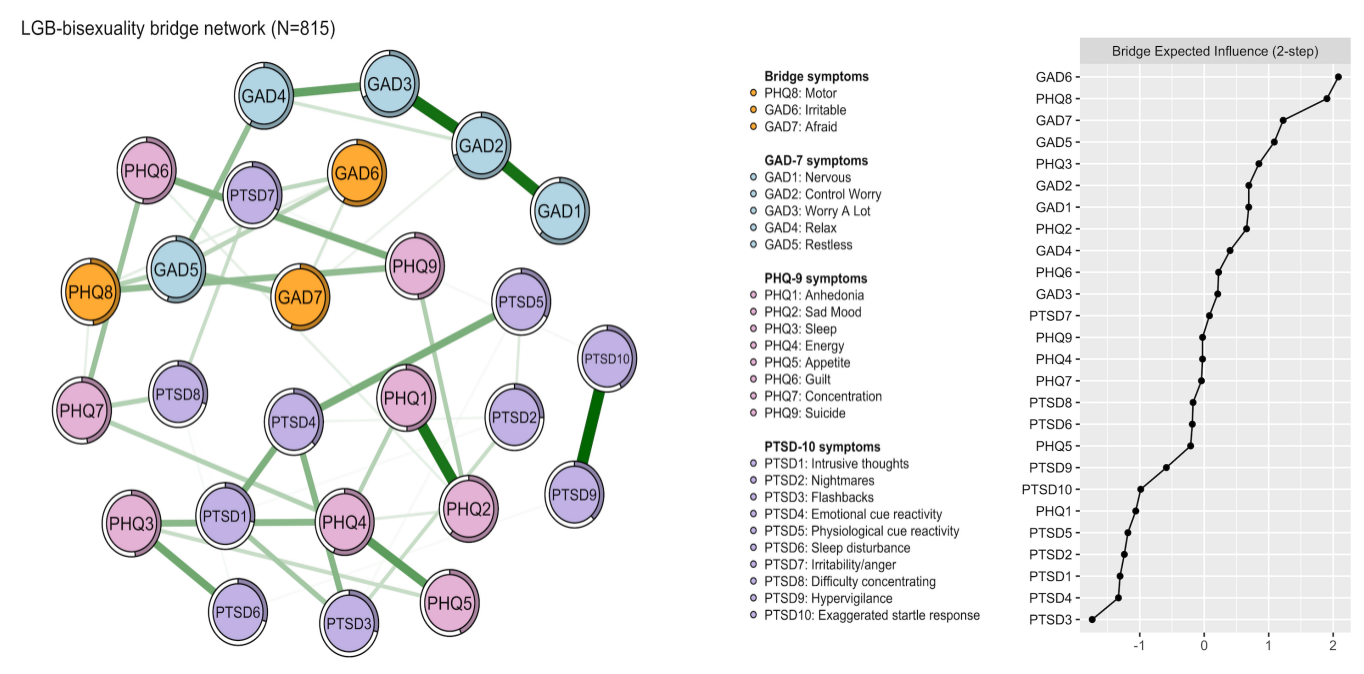


Note: GAD, the seven-item Generalized Anxiety Disorders Scale; PHQ, the nine-item Patient Health Questionnaire; PTSD, measured by the ten-item Trauma Screening Questionnaire.

Figure S7. The network structure of bridge symptoms of bullied other sexual minority youth (N = 569).


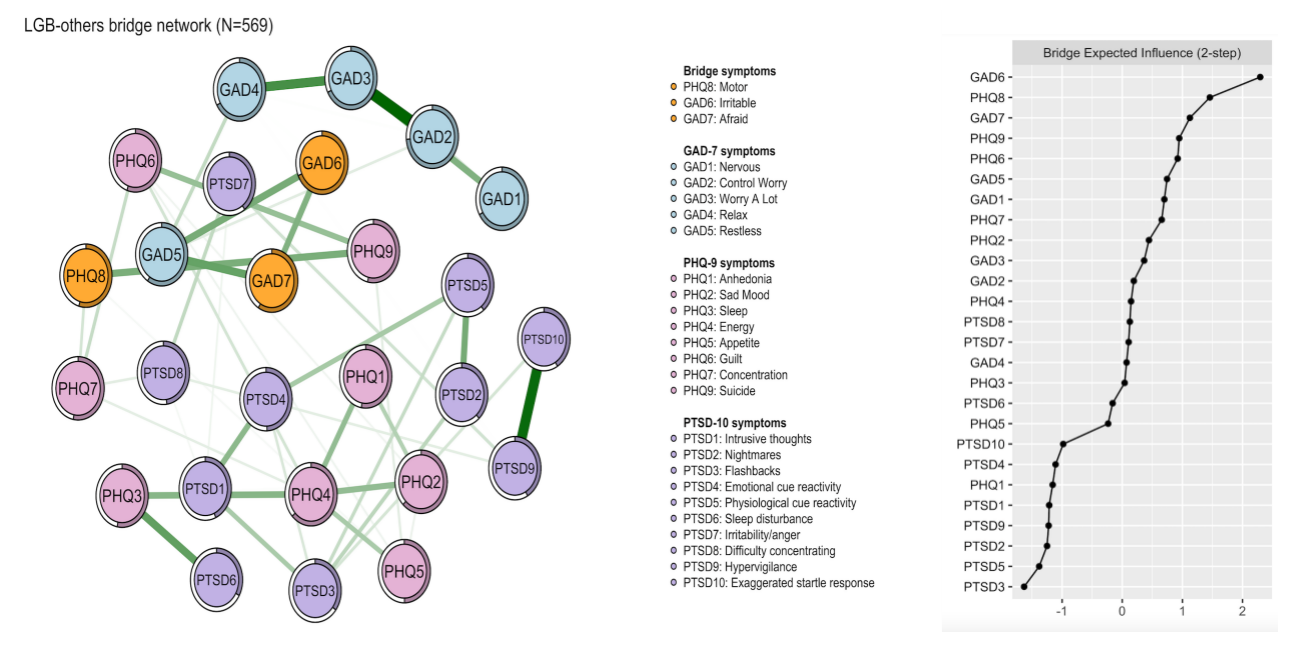


Note: GAD, the seven-item Generalized Anxiety Disorders Scale; PHQ, the nine-item Patient Health Questionnaire; PTSD, measured by the ten-item Trauma Screening Questionnaire.

Figure S8. Bridge strength, bridge betweenness, bridge closeness of networks of anxiety, depression, and post-traumatic stress disorder among all SMY and SMY subgroups.


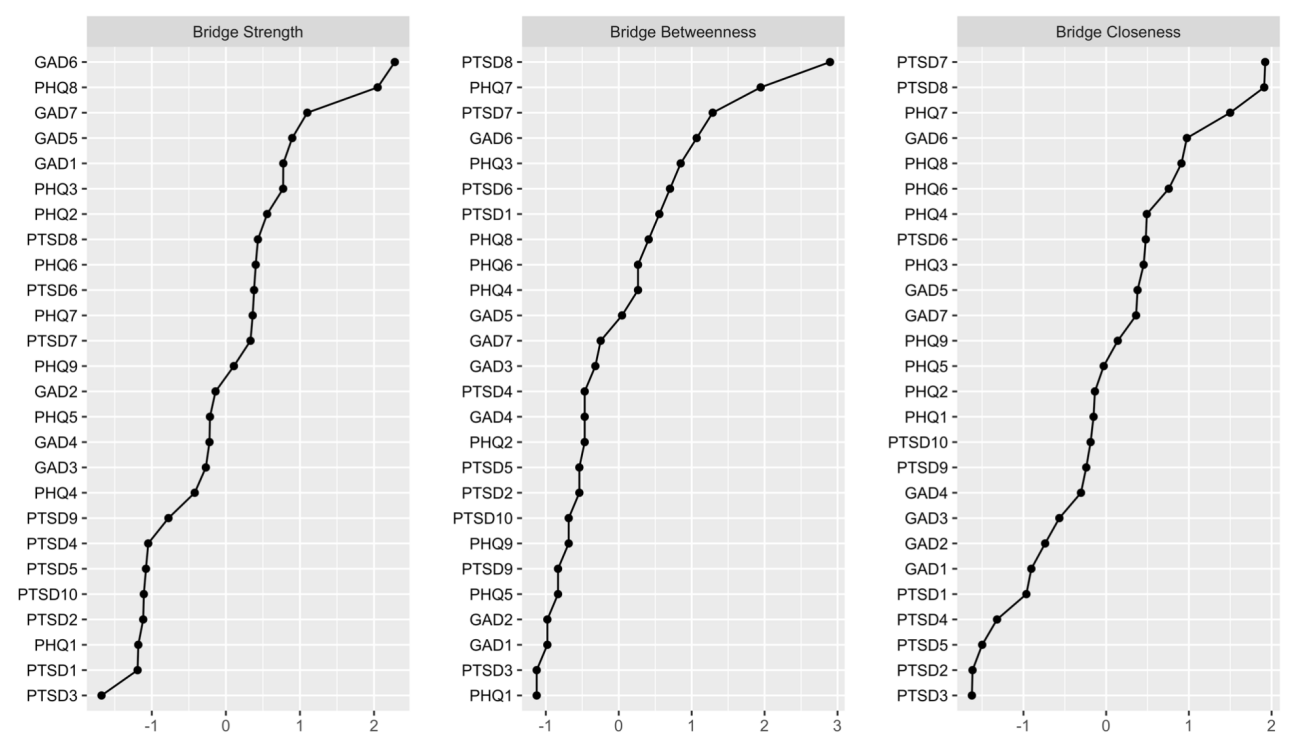

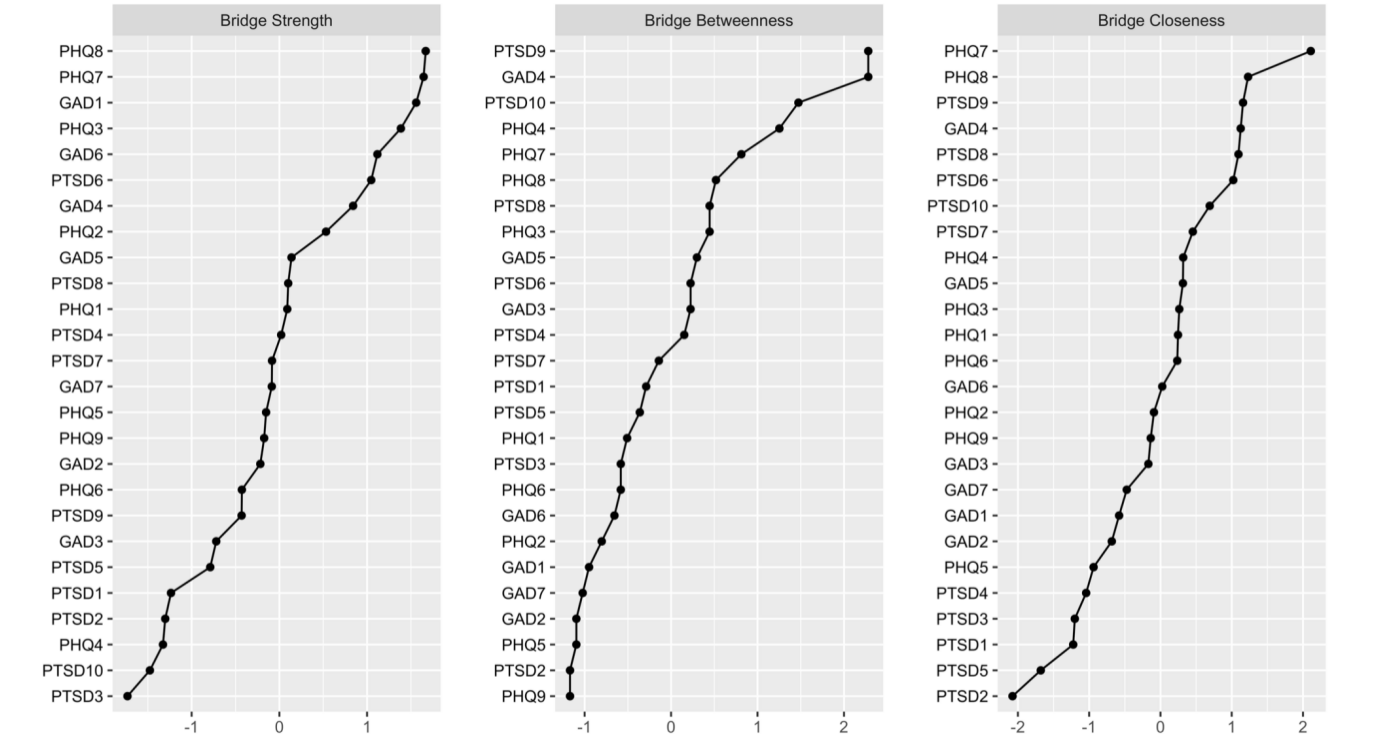


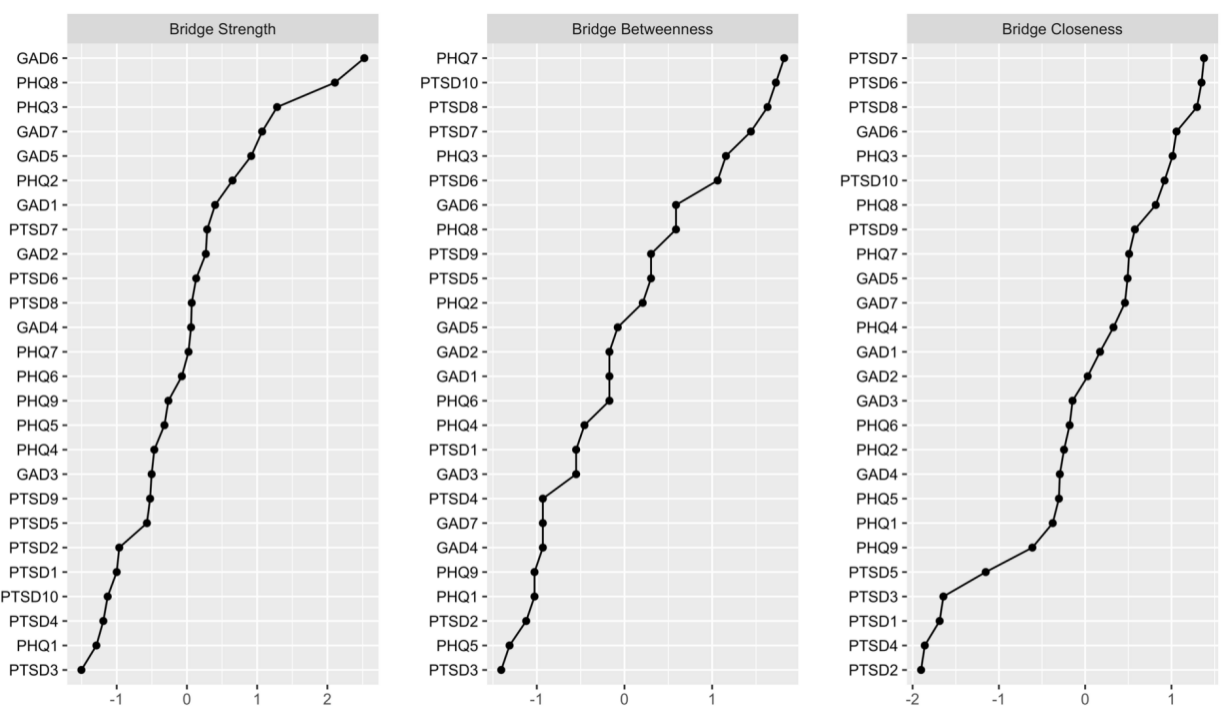

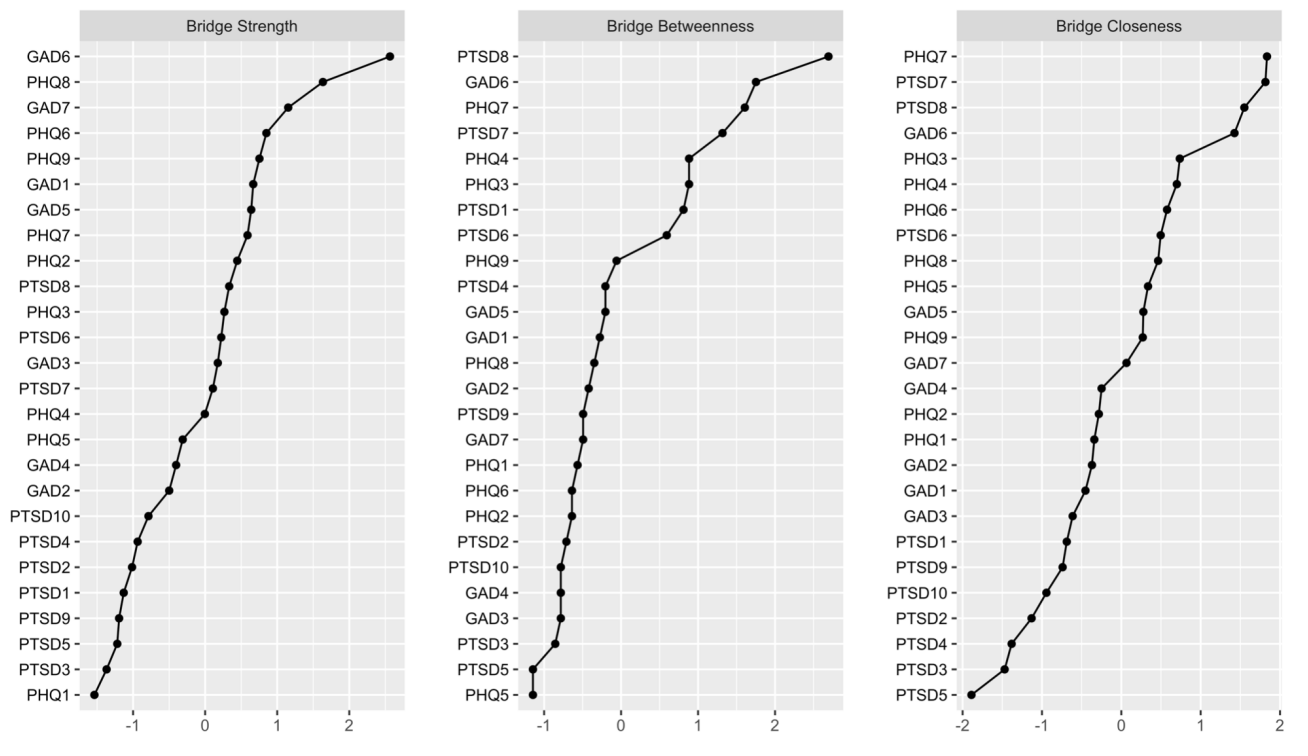


Note: SMY, sexual minority youth; GAD, the seven-item Generalized Anxiety Disorders Scale; PHQ, the nine-item Patient Health Questionnaire; PTSD, measured by the ten-item Trauma Screening Questionnaire.

Figure S9. The stability of centrality and bridge centrality indices of the global network using case-dropping bootstrap.


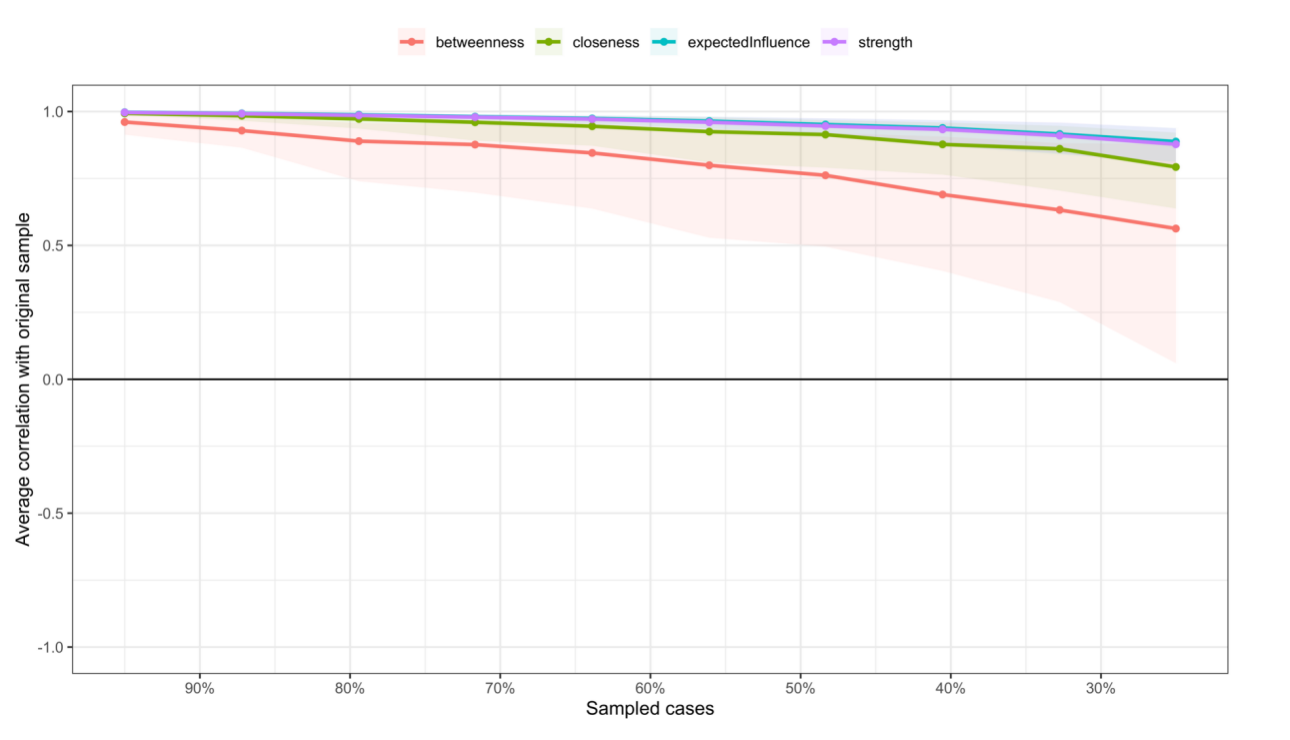

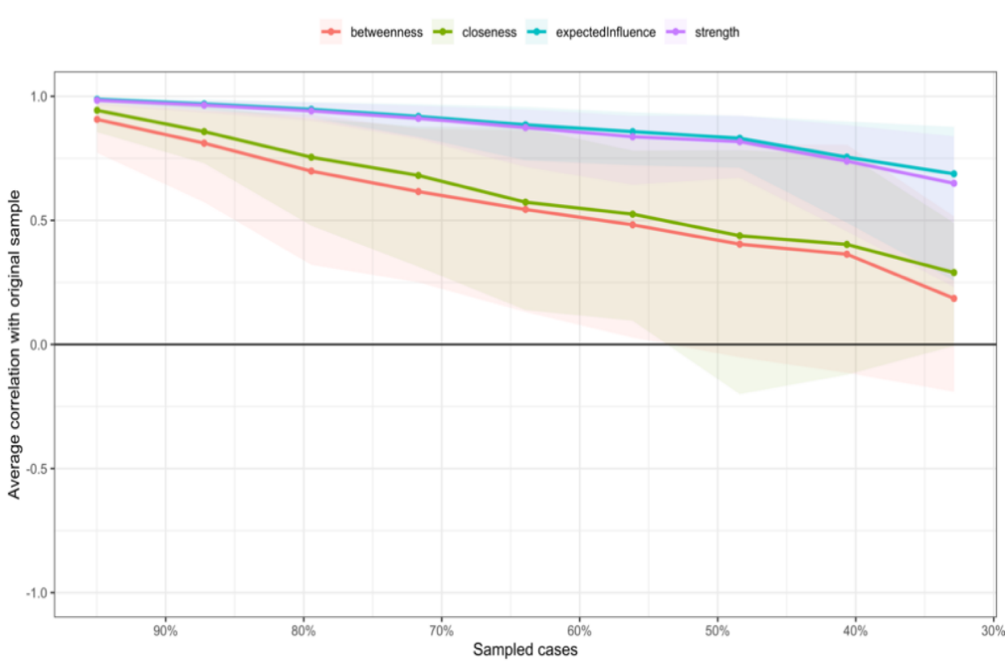


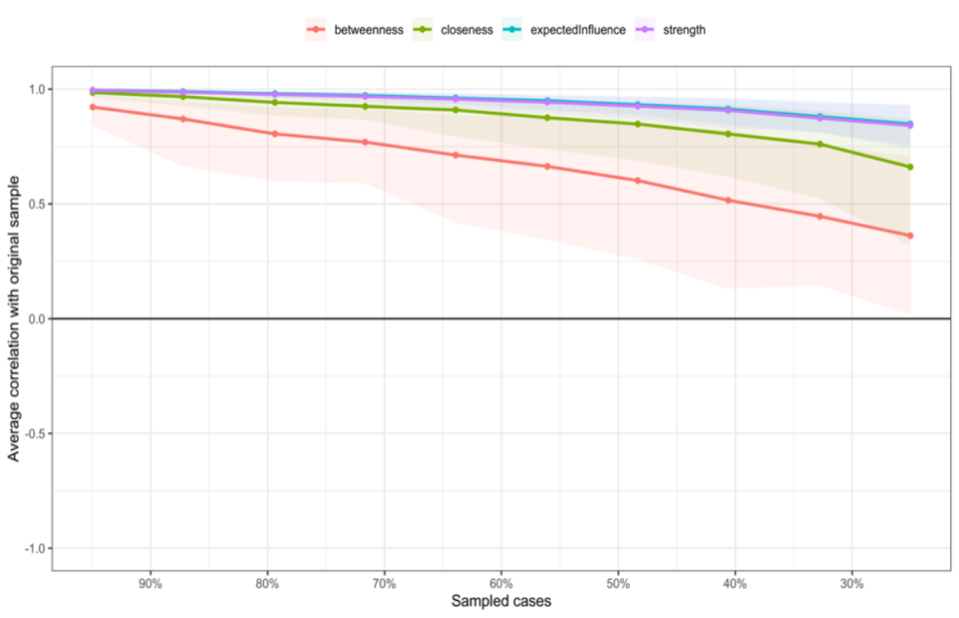

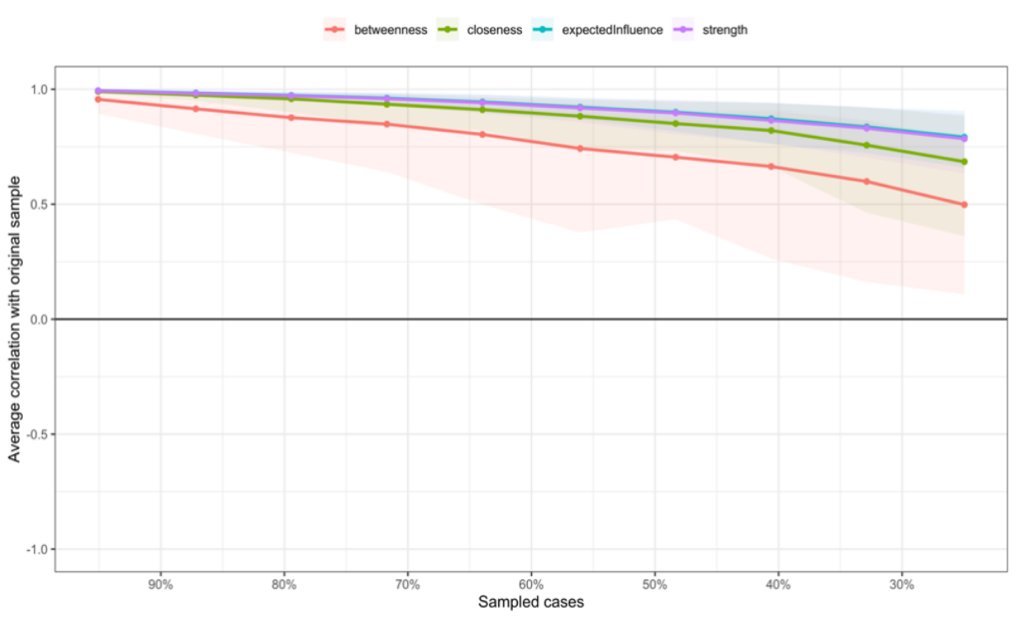


Figure S10. Bootstrapped confidence intervals of edge weights of global and subgroups’ networks.


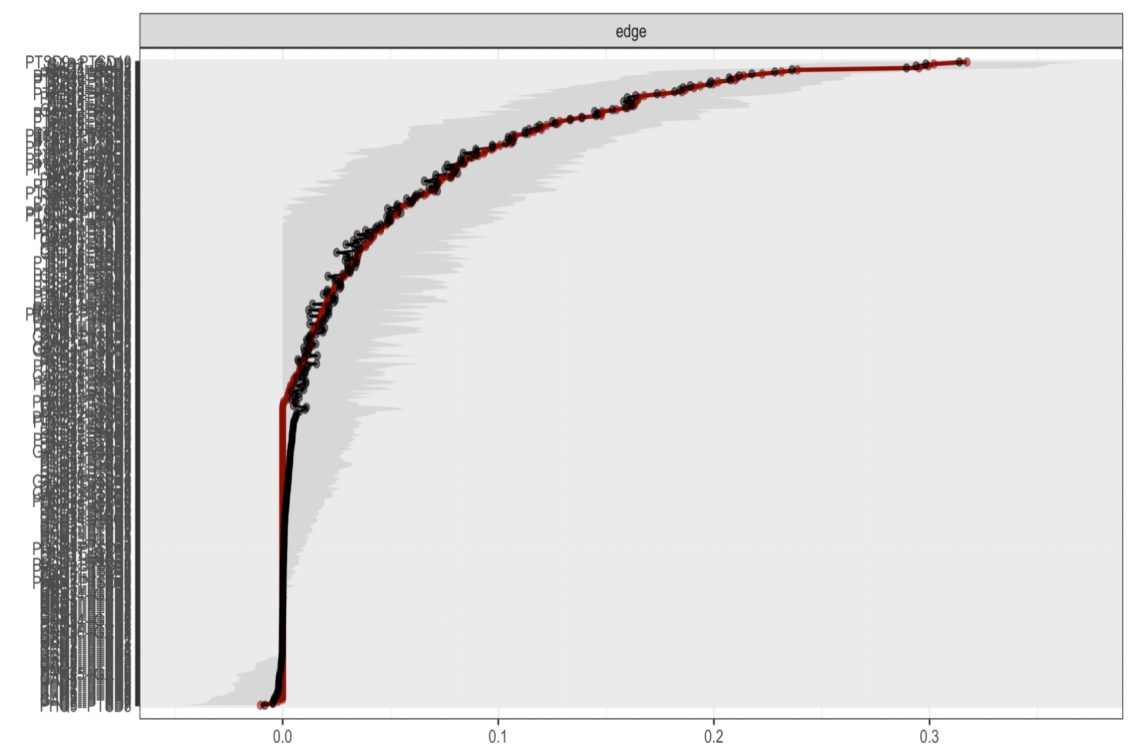

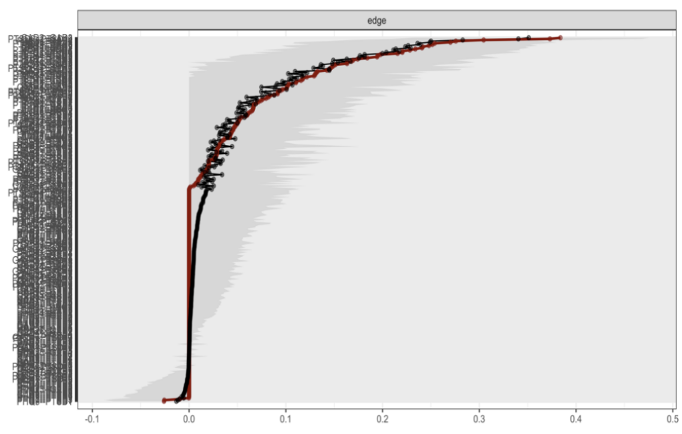

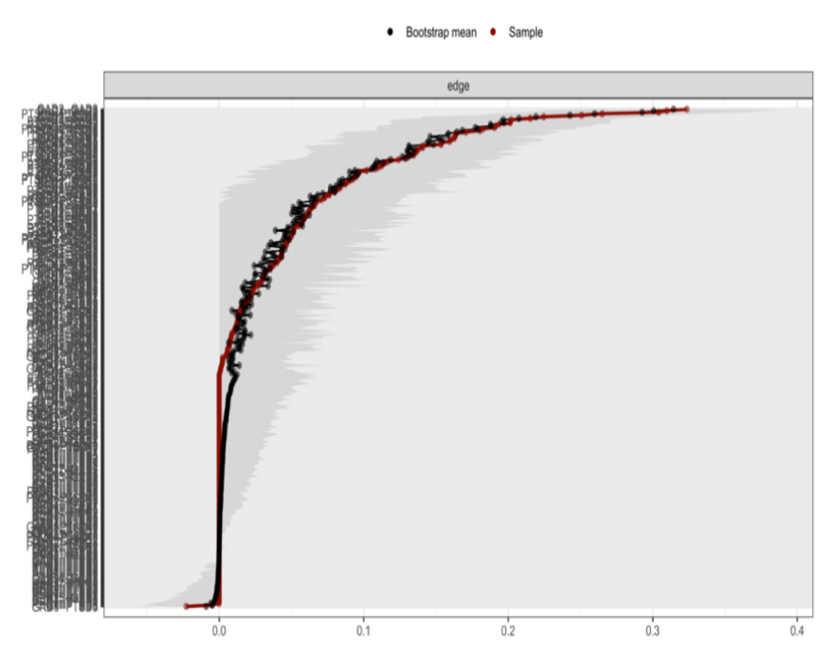

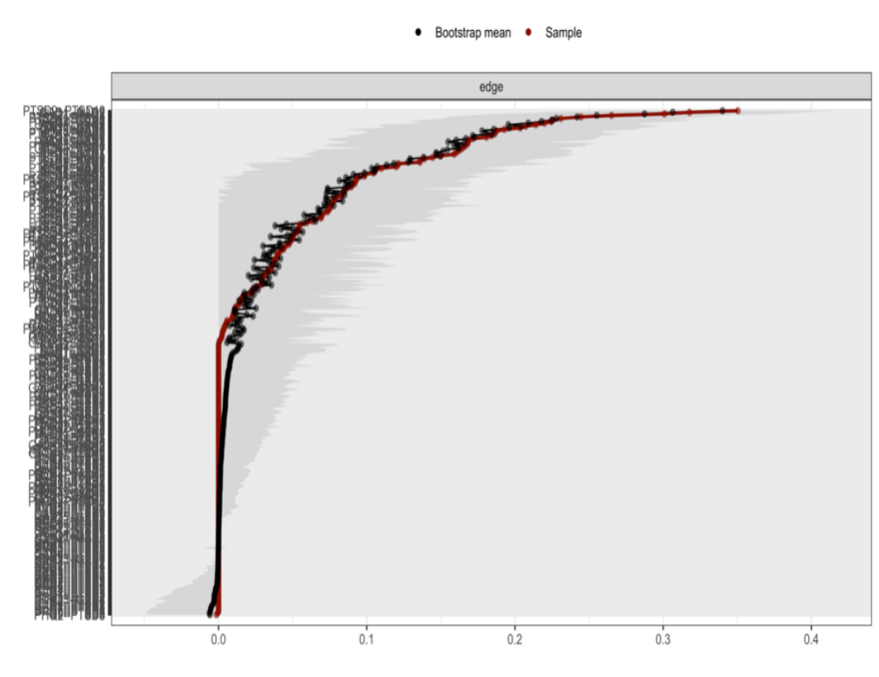


Figure S11. Estimation of edge weight difference of networks by bootstrapped difference test.


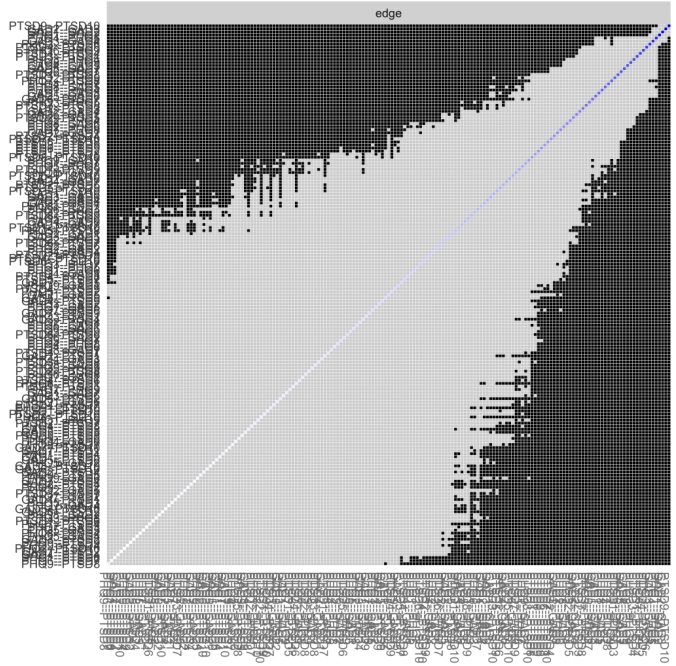

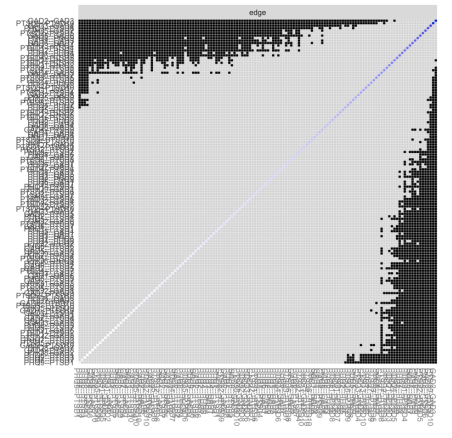


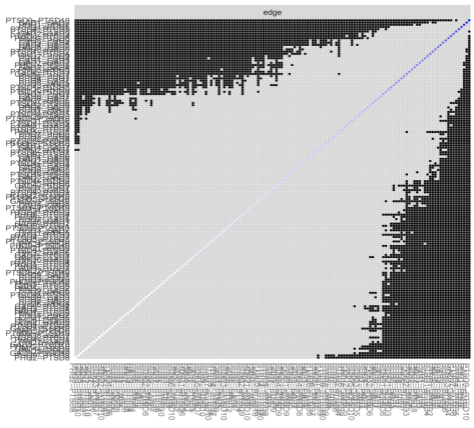

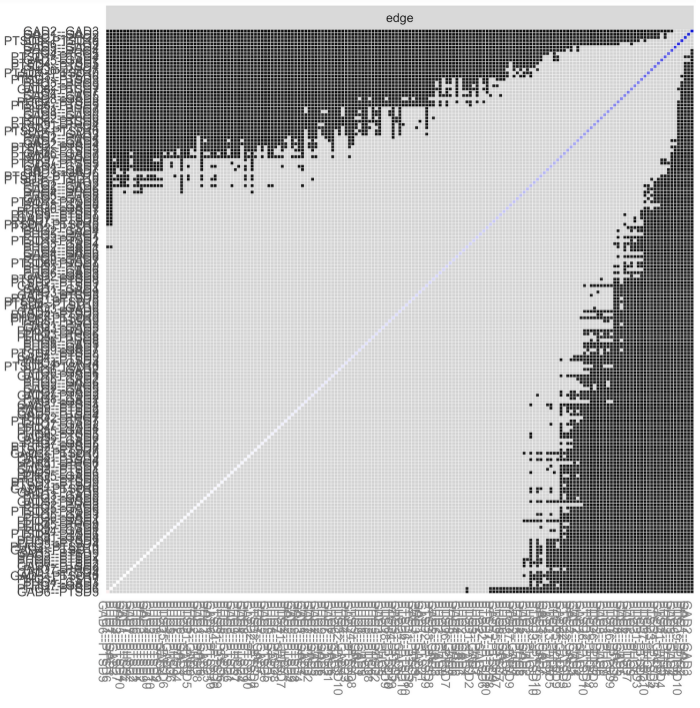


Figure S12. Estimation of strength difference of networks by bootstrapped difference test.


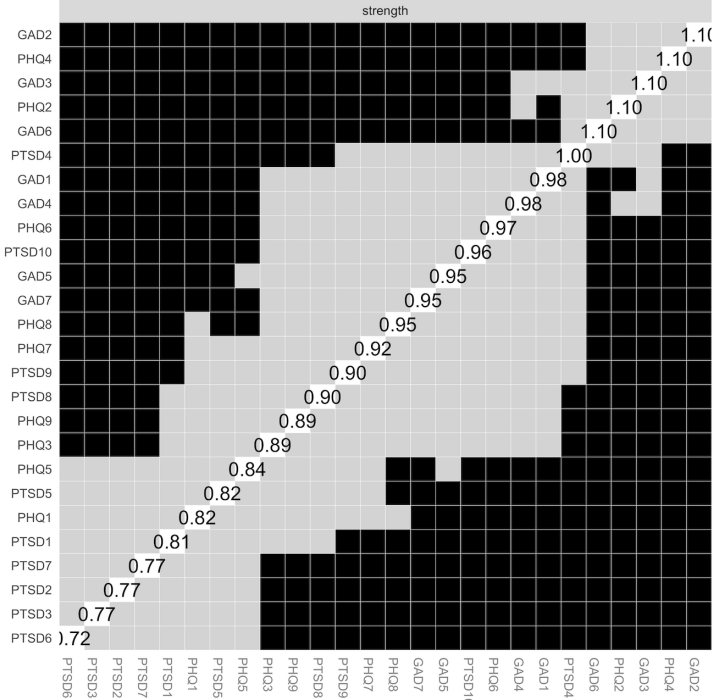

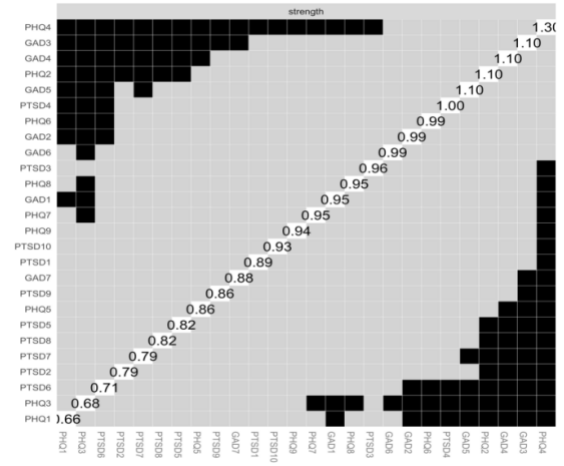


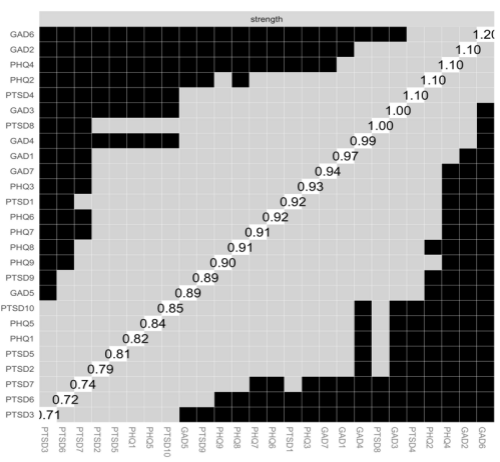

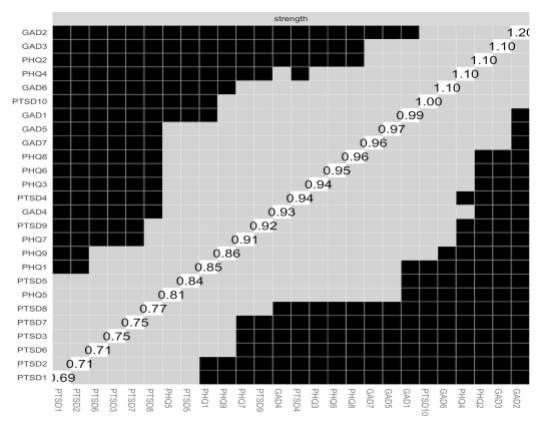


Figure S13. The Bayesian network of anxiety, depression, and post-traumatic stress disorder among bullied sexual minority youth based on a directed acyclic graph.


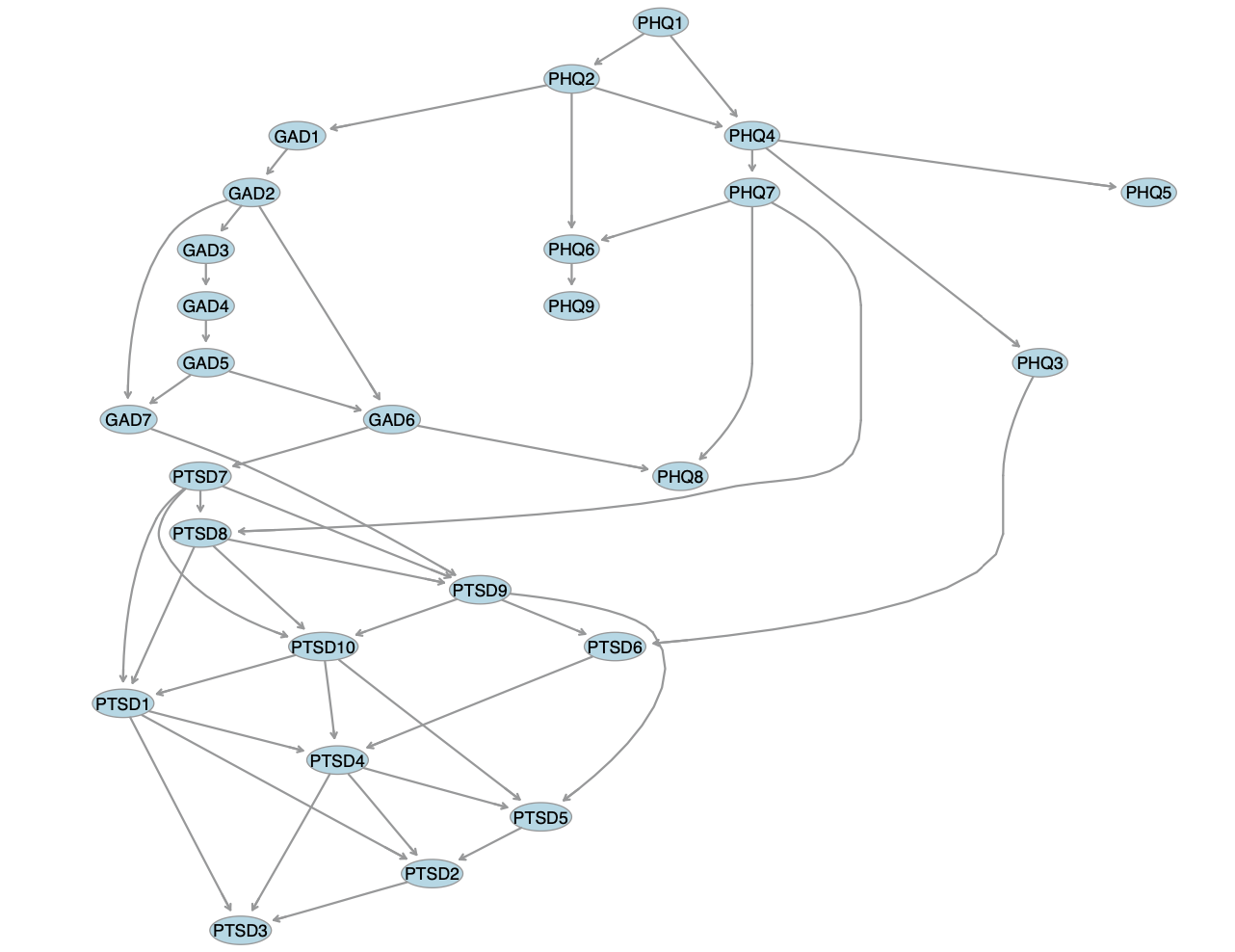


Note: GAD, the seven-item Generalized Anxiety Disorders Scale; PHQ, the nine-item Patient Health Questionnaire; PTSD, measured by the ten-item Trauma Screening Questionnaire.

Figure S14. The comparison of network structures among homosexual (Gay/Lesbian), bisexual, and other sexual minority (Others) youth who were bullied on college campuses.


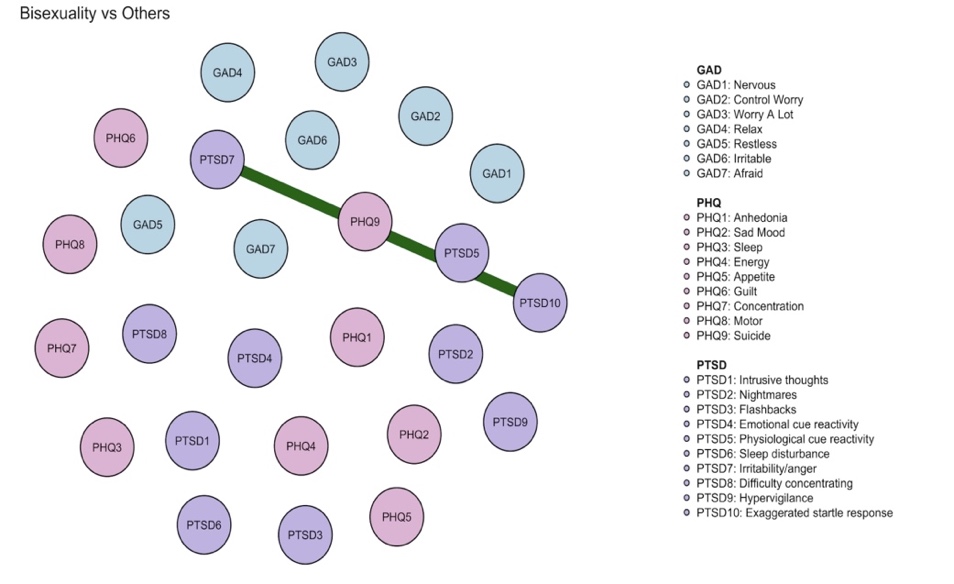

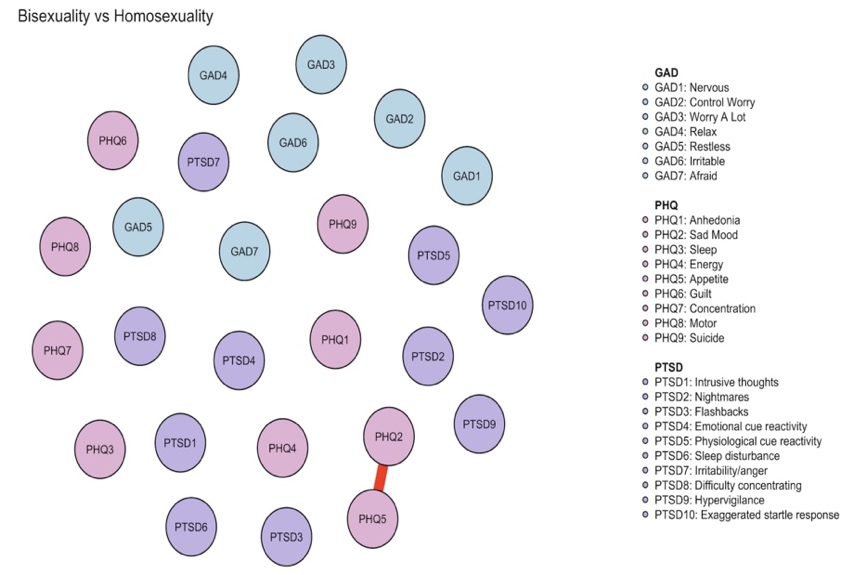


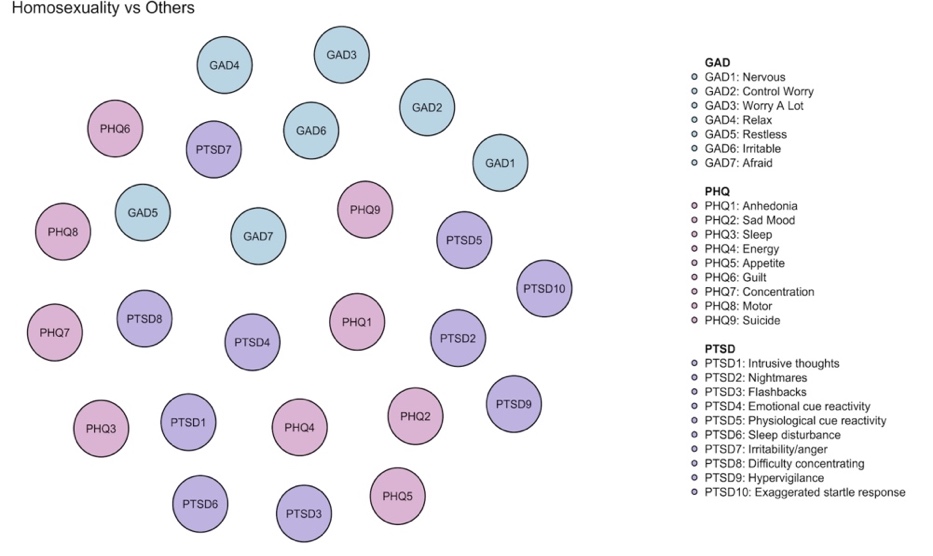


Note: GAD, the seven-item Generalized Anxiety Disorders Scale; PHQ, the nine-item Patient Health Questionnaire; PTSD, measured by the ten-item Trauma Screening Questionnaire.

**Supplementary References**

1. Garnets LD. Sexual orientations in perspective. Cultur Divers Ethnic Minor Psychol. 2002 May;8(2):115-29. PMID: 11987589. doi: 10.1037/1099-9809.8.2.115.

2. TSER TSER. Definitions. 2020.

3. Kroenke K, Spitzer RL, Williams JBW. The PHQ-9. Journal of General Internal Medicine. 2001 2001/09/01;16(9):606-13. doi: 10.1046/j.1525-1497.2001.016009606.x.

4. Wang W, Bian Q, Zhao Y, Li X, Wang W, Du J, et al. Reliability and validity of the Chinese version of the Patient Health Questionnaire (PHQ-9) in the general population. General hospital psychiatry. 2014 Sep-Oct;36(5):539-44. PMID: 25023953. doi: 10.1016/j.genhosppsych.2014.05.021.

5. Zhang Y-L, Liang W, Chen Z-M, Zhang H-M, Zhang J-H, Weng X-Q, et al. Validity and reliability of Patient Health Questionnaire-9 and Patient Health Questionnaire-2 to screen for depression among college students in China. Asia-Pacific Psychiatry. 2013;5(4):268-75. doi: 10.1111/appy.12103.

6. Spitzer RL, Kroenke K, Williams JB, Lowe B. A brief measure for assessing generalized anxiety disorder: the GAD-7. Arch Intern Med. 2006 May 22;166(10):1092-7. PMID: 16717171. doi: 10.1001/archinte.166.10.1092.

7. X He, CB Li, J Qian, HS Cui, Wu W. Reliability and validity of a generalized anxiety disorder scale in general hospital outpatient. Shanghai Archives of Psychiatry. 2010;22(4):200-3.

8. Brewin CR, Rose S, Andrews B, Green J, Tata P, McEvedy C, et al. Brief screening instrument for post-traumatic stress disorder. British Journal of Psychiatry. 2002;181(2):158-62. doi: 10.1192/bjp.181.2.158.

9. Foa EB, Riggs DS, Dancu CV, Rothbaum BO. Reliability and validity of a brief instrument for assessing post-traumatic stress disorder. Journal of Traumatic Stress. 1993;6(4):459-73. doi: 10.1002/jts.2490060405.

10. Walters JTR, Bisson JI, Shepherd JP. Predicting post-traumatic stress disorder: validation of the Trauma Screening Questionnaire in victims of assault. Psychological Medicine. 2007;37(1):143-50. doi: 10.1017/S0033291706008658.

11. de Bont PA, van den Berg DP, van der Vleugel BM, de Roos C, de Jongh A, van der Gaag M, et al. Predictive validity of the Trauma Screening Questionnaire in detecting post-traumatic stress disorder in patients with psychotic disorders. Br J Psychiatry. 2015 May;206(5):408-16. PMID: 25792693. doi: 10.1192/bjp.bp.114.148486.

12. Zhang G-B, Wang G-F, Han AZ, Xu N, Xie G-D, Chen L-R, et al. Association between different stages of precollege school bullying and murder-related psychological behaviors among college students in Anhui Province, China. Psychiatry Research. 2019 2019/12/01/;282:112593. doi: [10.1016/j.psychres.2019.112593](https://doi.org/10.1016/j.psychres.2019.112593).

13. Ihaka R, Gentleman R. R: A Language for Data Analysis and Graphics. Journal of Computational and Graphical Statistics. 1996 1996/09/01;5(3):299-314. doi: 10.1080/10618600.1996.10474713.

14. Epskamp S, Borsboom D, Fried EI. Estimating psychological networks and their accuracy: A tutorial paper. Behavior Research Methods. 2018 2018/02/01;50(1):195-212. doi: 10.3758/s13428-017-0862-1.

15. Robinaugh DJ, Millner AJ, McNally RJ. Identifying highly influential nodes in the complicated grief network. J Abnorm Psychol. 2016 Aug;125(6):747-57. PMID: 27505622. doi: 10.1037/abn0000181.

16. Jones PJ, Ma R, McNally RJ. Bridge Centrality: A Network Approach to Understanding Comorbidity. Multivariate Behav Res. 2021 Mar-Apr;56(2):353-67. PMID: 31179765. doi: 10.1080/00273171.2019.1614898.

17. Haslbeck JMB, Waldorp LJ. mgm: Estimating Time-Varying Mixed Graphical Models in High-Dimensional Data. Journal of Statistical Software. 2020 04/27;93(8):1 - 46. doi: 10.18637/jss.v093.i08.

18. van Borkulo CD, van Bork R, Boschloo L, Kossakowski JJ, Tio P, Schoevers RA, et al. Comparing network structures on three aspects: A permutation test. Psychol Methods. 2022 Apr 11. PMID: 35404628. doi: 10.1037/met0000476.

19. Briganti G, Scutari M, McNally RJ. A tutorial on bayesian networks for psychopathology researchers. Psychol Methods. 2022 Feb 3. PMID: 35113632. doi: 10.1037/met0000479.

20. Digitale JC, Martin JN, Glymour MM. Tutorial on directed acyclic graphs. J Clin Epidemiol. 2022 Feb;142:264-7. PMID: 34371103. doi: 10.1016/j.jclinepi.2021.08.001.

21. Scutari M. Learning Bayesian networks with the bnlearn R Package. Journal of Statistical Software. 2010;35(3):1–22. doi: 10.48550/arXiv.0908.3817.

22. Castelletti F, Consonni G, Della Vedova ML, Peluso S. Learning Markov Equivalence Classes of Directed Acyclic Graphs: An Objective Bayes Approach. Bayesian Analysis. 2018;13(4):1235-60, 26. doi: 10.1214/18-BA1101.

23. Kalisch M, Mächler M, Colombo D, Maathuis MH, Bühlmann P. Causal Inference Using Graphical Models with the R Package pcalg. Journal of Statistical Software. 2012 05/17;47(11):1 - 26. doi: 10.18637/jss.v047.i11.
